# Functional competence of a partially engaged GPCR–β-arrestin complex

**DOI:** 10.1038/ncomms13416

**Published:** 2016-11-09

**Authors:** Punita Kumari, Ashish Srivastava, Ramanuj Banerjee, Eshan Ghosh, Pragya Gupta, Ravi Ranjan, Xin Chen, Bhagyashri Gupta, Charu Gupta, Deepika Jaiman, Arun K. Shukla

**Affiliations:** 1 Department of Biological Sciences and Bioengineering, Indian Institute of Technology, Kanpur 208016, India; 2 School of Pharmaceutical Engineering and Life Sciences, Changzhou University, Changzhou, Jiangsu 213164, China

## Abstract

G Protein-coupled receptors (GPCRs) constitute the largest family of cell surface receptors and drug targets. GPCR signalling and desensitization is critically regulated by β-arrestins (βarr). GPCR–βarr interaction is biphasic where the phosphorylated carboxyl terminus of GPCRs docks to the N-domain of βarr first and then seven transmembrane core of the receptor engages with βarr. It is currently unknown whether fully engaged GPCR–βarr complex is essential for functional outcomes or partially engaged complex can also be functionally competent. Here we assemble partially and fully engaged complexes of a chimeric β_2_V_2_R with βarr1, and discover that the core interaction is dispensable for receptor endocytosis, ERK MAP kinase binding and activation. Furthermore, we observe that carvedilol, a βarr biased ligand, does not promote detectable engagement between βarr1 and the receptor core. These findings uncover a previously unknown aspect of GPCR-βarr interaction and provide novel insights into GPCR signalling and regulatory paradigms.

## Abstract (web summary)

β-arrestins initially contact with the phosphorylated carboxyl-terminus of GPCRs before engaging with the GPCR core. Here, the authors use a chimeric GPCR partially and fully engaged with β-arrestin1 and show that the core interaction is dispensable for receptor endocytosis and signalling.

## Graphical abstract

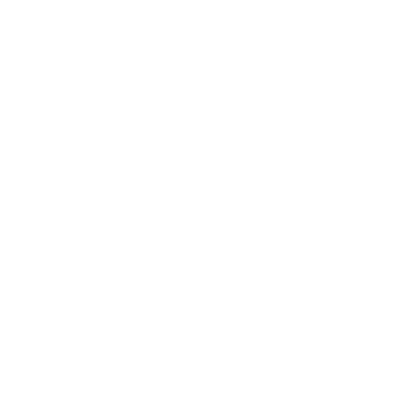

G protein-coupled receptor (GPCR) family consists of ∼800 different members that exhibit a highly conserved seven transmembrane architecture^1^. GPCRs bind to an incredibly diverse range of ligands, still, their signalling and regulatory mechanisms are primarily conserved^2^. GPCR signalling and downregulation is critically mediated by βarrs which on one hand, terminate G protein coupling presumably by steric hindrance and on the other, initiate G protein independent signalling cascades^3^. There has been a remarkable progress in structural visualization of GPCRs in the recent years^4^. However, structural details of GPCR–βarr interaction have just started to emerge and still remain in infancy. Interaction of the N-domain of arrestins with phosphorylated carboxyl terminus of GPCRs is the first step in receptor-arrestin binding. Interestingly, a number of biophysical studies using spectroscopy approaches have suggested the engagement of different arrestin loops with the activated receptor core as the second step of interaction^5^,^6^,^7^,^8^. Crystal structure of rhodopsin with isolated finger loop peptide has directly established a binding interface between the receptor core and the finger loop of visual arrestin^9^. Recently determined crystal structure of rhodopsin-arrestin complex also exhibits an engagement of the receptor core with arrestin^10^ although the carboxyl terminus of rhodopsin in this complex is covalently fused and not phosphorylated. Recent visualization of β_2_V_2_R–βarr1 complex by negative stain electron microscopy and cross-linking has directly demonstrated a biphasic mechanism of GPCR–βarr interaction^11^. In the first step, the phosphorylated carboxyl terminus of GPCRs interacts with the N-domain of βarrs and in the second step, βarrs engage with the cytoplasmic surface of the transmembrane bundle of the receptor (that is, receptor core) (Fig. 1a).

The functional repertoire of GPCR-βarr signalling axis is quite broad and spans a wide range of cellular and physiological processes^3^,^12^,^13^,^14^. This is primarily mediated by a large number of interactions of βarrs and their abilities to scaffold a wide array of kinases and other signalling molecules^12^,^13^. However, the structural and mechanistic requirements for such a broad functional coverage of GPCR–βarr interaction remains currently unexplored. In particular, whether a fully engaged GPCR–βarr complex is essential for triggering downstream functional outcomes or even partially engaged complexes might display functional competence remains currently unknown. Phosphorylation of the carboxyl terminus of GPCRs is the primary determinant for βarr interaction and this first step of biphasic interaction represents the high-affinity component in GPCR–βarr complex^15^,^16^,^17^. Direct visualization of a partially engaged β_2_V_2_R–βarr1 complex^11^ associated solely through the phosphorylated carboxyl terminus of the receptor by electron microscopy suggests that core interaction may be dispensable for stable assembly of the complex. However, functional capabilities of such a partially engaged receptor–βarr complex remain currently unexplored.

Accordingly, here we set out to investigate whether a β_2_V_2_R–βarr1 complex associated only through the phosphorylated carboxyl terminus of the receptor and lacking the core interaction might be functionally competent. We focus on recruitment and activation of ERK (extracellular signal-regulated kinase) MAP (mitogen-activated protein) kinase, a readout that has become quintessential for βarr mediated GPCR signalling, and receptor endocytosis. We assemble partially and fully engaged β_2_V_2_R–βarr1 complexes, validate them by fluorescence spectroscopy and discover, in contrast with generally believed notion, that the core interaction in this complex is dispensable for ERK2 binding and activation. We also find that a receptor mutant lacking the core interaction with βarr efficiently undergoes agonist promoted internalization. Moreover, we also discover that a βarr biased ligand does not promote core interaction between the receptor and βarr.

## Results

### Partially and fully engaged β_2_V_2_R–βarr1 complexes

**Figure 1:**
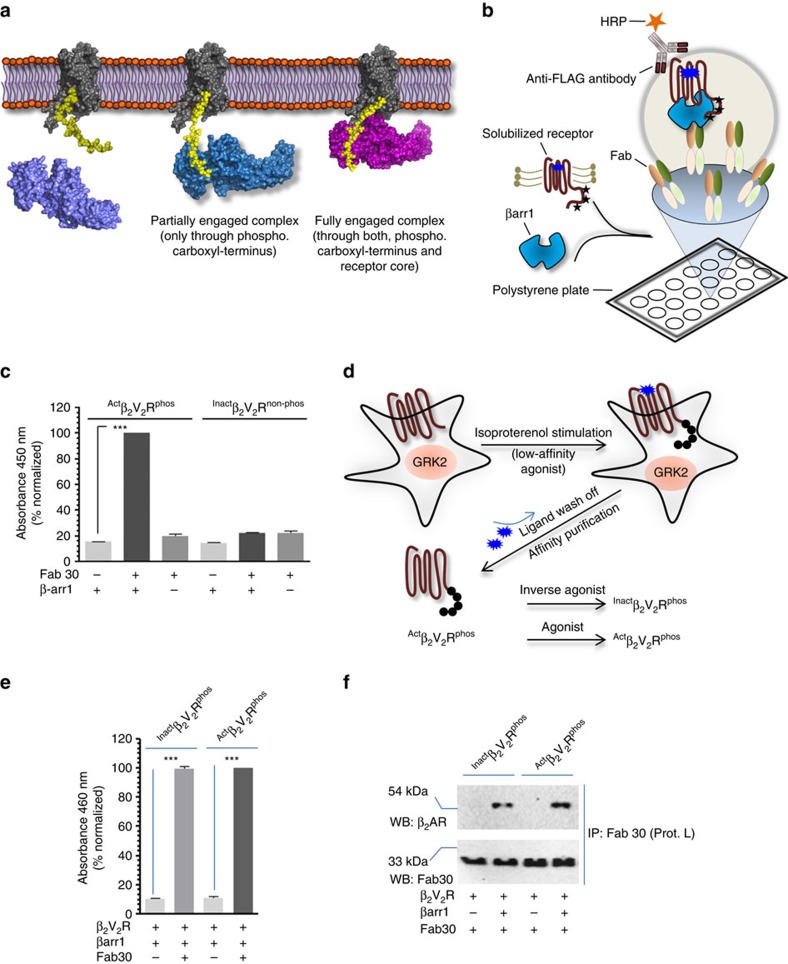
Assembly of partially and fully engaged β_2_V_2_R–βarr1-Fab30 complex. (**a**) Schematic representation of biphasic GPCR–βarr interaction. βarr interacts with activated and phosphorylated GPCRs in a biphasic fashion where the first step is binding of βarr through the phosphorylated carboxyl terminus and the second step is the engagement of βarr with the 7TM core of the receptor. The receptor component is shown in grey, phosphorylated carboxyl terminus in yellow and βarr 1 in blue/magenta. (**b**) Schematic representation of an ELISA-based approach for *in-vitro* assembly of β_2_V_2_R–βarr1 complex. Purified Fab30 is immobilized on solid support as an anchor to capture the complex followed by incubation with purified β_2_V_2_R and βarr1. Formation of β_2_V_2_R–βarr1 complex is visualized using HRP-coupled anti-FLAG M2 antibody through detection of FLAG tagged β_2_V_2_R. (**c**) Fab 30 assisted *in-vitro* assembly of β_2_V_2_R–βarr1 complex. Agonist bound and phosphorylated β_2_V_2_R (^Act^β_2_V_2_R^phos^) forms a stable complex while inverse agonist bound and non-phosphorylated β_2_V_2_R (^Inact^β_2_V_2_R^non-phos^) does not exhibit any detectable complex formation. (**d**) An experimental set-up to assemble ‘tail only' engaged and ‘fully' engaged β_2_V_2_R–βarr1 complex *in-vitro*. β_2_V_2_R is coexpressed with GRK2^CAAX^ in cultured *Sf*9 cells and 66 h post-infection, cells are stimulated with a low-affinity agonist (Isoproterenol) to trigger receptor phosphorylation. Subsequently, the receptor is purified by affinity chromatography and the ligand is washed off during purification to yield ligand free phosphorylated β_2_V_2_R (^Apo^β_2_V_2_R^phos^). Incubation with inverse agonist (carazolol) or high-affinity full agonist (BI-167107) yields ^Inact^β_2_V_2_R^phos^ and ^Act^β_2_V_2_R^phos^, respectively. (**e**) Both, the ^Inact^β_2_V_2_R^phos^ and ^Act^β_2_V_2_R^phos^ form a stable complex with βarr1 as assessed by ELISA approach and potentially represent ‘tail only' and ‘fully' engaged complexes, respectively. (**f**) Formation of ‘tail only' engaged and ‘fully' engaged complexes as assessed by coimmunoprecipitation experiment. This experiment was repeated three times with identical results and a representative image is shown. Signals in **c** and **e** are normalized with ^Act^β_2_V_2_R^phos^+βarr1+Fab30 condition as 100%. Data presented in **c** and **e** represent mean±s.e.m. of three independent experiments each carried out in duplicate and analysed using one-way ANOVA with Bonferroni post-test (***P<0.001).

Reconstitution of a stable and functional GPCR–βarr complex for biophysical studies still remains very challenging. Recently, a strategy has been described for the isolation of a stable βarr1 complex with a chimeric β_2_ adrenergic receptor (β_2_AR) harbouring the carboxyl terminus of the arginine vasopressin subtype 2 receptor (V_2_R), referred to as β_2_V_2_R (ref. 11). β_2_V_2_R displays β_2_AR pharmacology but tighter binding with βarr^18^. Stable β_2_V_2_R–βarr1 complex can be isolated through coexpression of the receptor and βarr1 in cells followed by stabilization using a synthetic antibody fragment (referred to as Fab30) (ref. 11). In order to make this strategy more versatile and amenable to direct biophysical studies, we first assessed the feasibility of β_2_V_2_R–βarr1-Fab30 complex assembly using purified components *in-vitro* (Fig. 1b). We immobilized purified Fab30 (Supplementary Fig. 1 and Supplementary Fig. 2A) on a polystyrene surface (MaxiSorp 96 well plate) as an anchor to stabilize the complex followed by addition of purified βarr1 and N-terminally FLAG-tagged β_2_V_2_R (Supplementary Fig. 2B and Supplementary Fig. 3). After rigorous washing of the surface, we visualized the assembly of the complex using HRP-coupled anti-FLAG M2 antibody. We observed a robust assembly of β_2_V_2_R–βarr1 complex that is sensitive to agonist occupancy and phosphorylation status of the receptor, suggesting the formation of a cellularly and pharmacologically relevant complex (Fig. 1c and Supplementary Fig. 2C–E).

As mentioned earlier, in biphasic GPCR–βarr interaction, the first step depends primarily on phosphorylation of carboxyl terminus of the receptor while the second step requires an activated receptor core (that is, transmembrane bundle). Therefore, in order to generate partially and fully engaged complexes, we designed an experimental scheme (Fig. 1d) where we trigger receptor phosphorylation in cells by stimulating them with a low-affinity full agonist, isoproterenol and then wash off the agonist in subsequent purification steps. This leads to purification of ligand free β_2_V_2_R with phosphorylated carboxyl terminus (referred to as ^Apo^β_2_V_2_R^phos^). Subsequent incubation with high-affinity partial inverse agonist (carazolol) or high-affinity full agonist (BI-167107) results in ^Inact^β_2_V_2_R^phos^ (inactive receptor core with phosphorylated carboxyl terminus) and ^act^β_2_V_2_R^phos^ (active receptor core with phosphorylated carboxyl terminus), respectively. These two species of the β_2_V_2_R provide us a handle to assemble partially (that is, tail only engaged) and fully (that is, tail+core engaged) associated β_2_V_2_R–βarr1 complexes and evaluate their functional competence *in-vitro*. As presented in Fig. 1e,f, both, the ^Inact^β_2_V_2_R^phos^ and the ^act^β_2_V_2_R^phos^ exhibited robust complex formation with βarr1 and presumably represent, partially and fully engaged β_2_V_2_R–βarr1 complexes, respectively.

**Figure 2:**
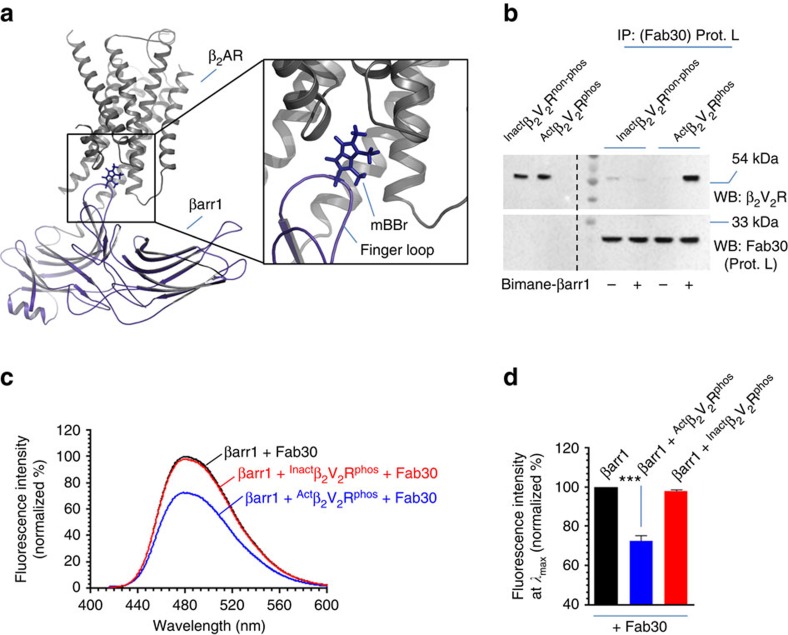
Validation of partially and fully engaged complexes by fluorescence spectroscopy. (**a**) Structural model of β_2_AR–β-arr1 complex deduced based on negative-stain electron microscopy, cross-linking experiments and hydrogen-deuterium exchange mass-spectrometry reveals finger loop of βarr1 as a key component of the core interaction. L^68^ in the finger loop of βarr1 was changed to cysteine in a cysteine-less βarr1 and monobromobimane was attached to this cysteine by chemical coupling. Upon core interaction, bimane fluorescence intensity decreases either due to change in chemical environment or quenching by a tyrosine/tryptophan residue in the vicinity. (**b**) Functional validation of bimane labelled βarr1 by its interaction with purified β_2_V_2_R. Similar to wild-type βarr1, bimane labelled βarr1 also forms a complex with agonist occupied and phosphorylated β_2_V_2_R. The experiment was repeated twice with identical results and a representative image is shown. (**c**) Incubation of ^Act^β_2_V_2_R^phos^ but not ^Inact^β_2_V_2_R^phos^ with bimane labelled βarr1 leads to a decrease in bimane fluorescence. Considering equivalent physical interaction of ^Act^β_2_V_2_R^phos^ and ^Inact^β_2_V_2_R^phos^ (as presented in Fig. 1e,f), bimane fluorescence data suggests that ^Act^β_2_V_2_R^phos^ engages the core interaction while the ^Inact^β_2_V_2_R^phos^ does not. These data suggest that ^Inact^β_2_V_2_R^phos^+βarr1+Fab30 and ^Act^β_2_V_2_R^phos^+βarr1+Fab30 complexes represent ‘tail only' and ‘fully' (tail+core) engaged complexes, respectively. (**d**) Bimane fluorescence at emission *λ*_max_ as measured in **c** is presented as a bar graph. Data presented in **d** represent mean ±s.e.m. of three independent experiments analysed using one-way ANOVA with Bonferroni post-test (***P<0.001).

In order to confirm the nature of these complexes with respect to tail and core engagement, we utilized a bimane fluorescence spectroscopy approach. Extensive previous studies have used bimane labelling in the finger loop of visual arrestin to study its interaction with rhodopsin and reported that rhodopsin-arrestin interaction leads to a significant decrease in bimane fluorescence^5^,^9^,^19^,^20^. Direct engagement of finger loop of visual arrestin with rhodopsin has also been documented by NMR^21^ and crystallography^9^. Crystal structure of rhodopsin-arrestin complex also reveals an engagement of the finger loop of visual arrestin with the transmembrane core of rhodopsin^10^. More recently, chemical cross-linking and structural modelling of β_2_V_2_R–βarr1 complex has also identified the finger loop of βarr1 (residues 62-72) as a major interaction interface with the seven transmembrane core of the receptor^11^ (Fig. 2a). Therefore, we first designed a cysteine-less βarr1 mutant and then exchanged Leu^68^ in the finger loop with a cysteine (referred to as βarr^L68C^). We selected L^68^C based on previous studies with rhodopsin-visual arrestin system that have used the corresponding position (L^72^C) in the finger loop^5^,^6^,^19^,^20^,^22^. We subsequently purified βarr1^L68C^ and labelled it with an environmentally sensitive fluorophore monobromobimane (mBBr) at Cys^68^. Based on rhodopsin-arrestin studies, we reasoned that the environment of mBBr should change upon the engagement of the finger loop with the receptor core and, therefore, a change in mBBr fluorescence intensity will reflect the core interaction between β_2_V_2_R and βarr1. We confirmed the functionality of mBBr-labelled βarr1 with respect to its binding with agonist occupied and phosphorylated β_2_V_2_R by coimmunoprecipitation assay (Fig. 2b). We then tested β_2_V_2_R–βarr1-Fab30 complex by fluorescence spectroscopy and interestingly found that incubation of ^act^β_2_V_2_R^phos^ with mBBr-labelled βarr1 indeed resulted in a decrease in fluorescence intensity while that of ^Inact^β_2_V_2_R^phos^ does not (Fig. 2c,d). Considering that both ^Inact^β_2_V_2_R^phos^ and ^Act^β_2_V_2_R^phos^ interact with βarr1 comparably, this observation suggest that the complexes of βarr1 with ^Inact^β_2_V_2_R^phos^ and ^act^β_2_V_2_R^phos^ in fact represent, partially engaged (‘tail only') and fully engaged (‘tail+core') complexes, respectively.

**Figure 3:**
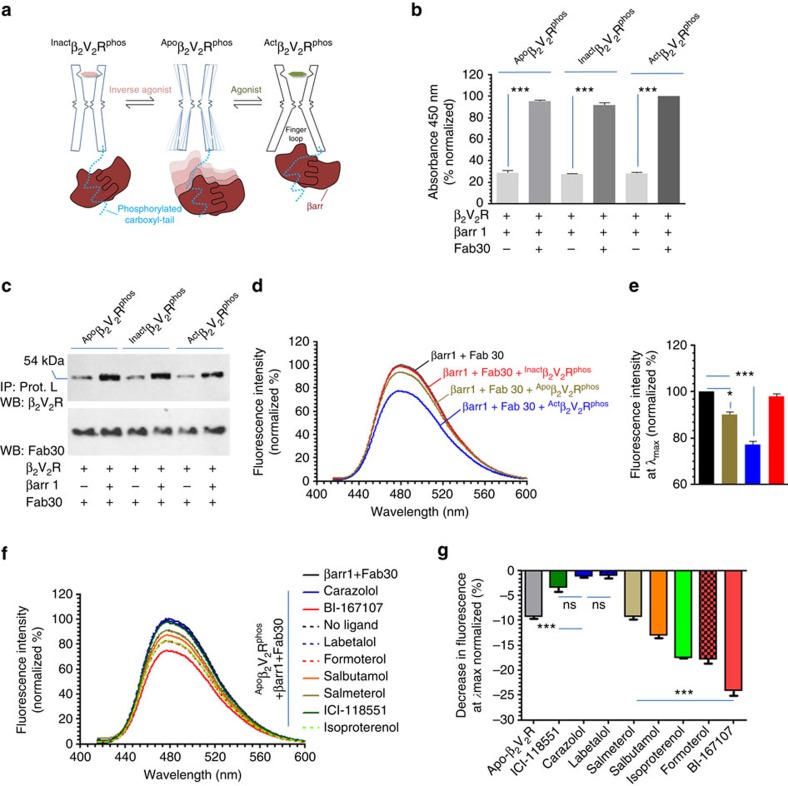
Ligand-dependent modulation of core interaction in ^Apo^β_2_V_2_R^phos^–βarr1-Fab30 complex. (**a**) A schematic representation showing that ^Apo^β_2_V_2_R^phos^ can potentially sample active like conformations and, therefore, might engage core interaction to some extent. Incubation with an inverse agonist is likely to ablate this basal level of core interaction yielding a ‘tail only' complex while incubation with an agonist stabilizes the core interaction and results in a ‘fully engaged' complex. (**b**) *In-vitro* assembly of ^Apo^β_2_V_2_R^phos^ complex with βarr1 in presence of Fab30 as assessed by ELISA approach. Incubation of this pre-formed complex with inverse agonist or agonist does not alter the physical assembly of the complex. (**c**) *In-vitro* assembly of ^Apo^β_2_V_2_R^phos^ complex with βarr1 in presence of Fab30 as measured by coimmunoprecipitation. Similar to ELISA approach, incubation of pre-formed complex with inverse agonist or agonist does not alter the complex assembly. This experiment was repeated three times with identical results and a representative image is shown. (**d**) Incubation of pre-formed ^Apo^β_2_V_2_R^phos^ complex with inverse agonist (carazolol) results in an increase in bimane fluorescence suggesting a loss of core binding, yet presumably stabilization of a ‘tail engaged' complex. On the other hand, incubation of this complex with agonist (BI-167107) results in a further decrease in bimane fluorescence suggesting the engagement of receptor core and, therefore, stabilization of a ‘fully engaged' complex. (**e**) Bimane fluorescence at emission *λ*_max_ as measured in **d** is presented as a bar graph. (**f**) Incubation of pre-formed ^Apo^β_2_V_2_R^phos^ complex with a panel of ligands results in different extent of bimane fluorescence quenching, which directly correlates to the ligand efficacy. (**g**) Quantification of decrease in bimane fluorescence at emission *λ*_max_ as measured in **f** is presented as a bar graph. Data in **d** and **f** represent mean of three independent experiments. Data presented in **b**, **e** and **g** represent mean±s.e.m. of three independent experiments and analysed using one-way ANOVA with Bonferroni post-test (*P<0.05; ***P<0.001).

In order to further confirm this, we used an alternative approach where we first assembled a complex of ^Apo^β_2_V_2_R^phos^ with βarr1 and then incubated it with either an inverse agonist or agonist to generate partially and fully engaged complexes, respectively. We reasoned that ^Apo^β_2_V_2_R^phos^ should form a complex with βarr1 primarily driven through the phosphorylated tail but it might also engage some core interaction owing to the constitutive activity of the receptor (Fig. 3a). We anticipated that incubation of this complex with inverse agonist should destabilize (and presumably ablate) the core interaction while agonist should further stabilize the core interaction. As presented in Fig. 3b,c, ^Apo^β_2_V_2_R^phos^ indeed forms a stable complex with βarr1, which is physically not altered by incubation with either the inverse agonist or agonist. Interestingly, however, bimane fluorescence level in ^Apo^β_2_V_2_R^phos^–βarr1-Fab30 complex was lower compared with βarr1(+Fab30), suggesting a basal level of core engagement in this complex (Fig. 3d,e). Incubation of this complex with agonist resulted in a robust decrease in fluorescence intensity suggesting the engagement of core interaction. On the other hand, incubation with inverse agonist led to an increase in bimane fluorescence bringing it up to βarr1 alone level indicating disengagement of basal core interaction (Fig. 3d,e).

In order to further corroborate that bimane fluorescence quenching is a reliable read out of core interaction, we tested a panel of receptor ligands with different efficacies on preformed ^Apo^β_2_V_2_R^phos^ complex. Again, incubation of pre-formed complex with these ligands does not alter the physical interaction as assessed by coimmunoprecipitation and enzyme-linked immunosorbent assay (ELISA) (Supplementary Fig. 4A–C). Strikingly, however, the degree of fluorescence quenching directly mirrors the ligand efficacy for the receptor (Fig. 3f,g). Furthermore, incubation of pre-formed complex with varying doses of the agonist (BI-167107) reveals that degree of fluorescence quenching directly corresponds to the ligand occupancy of the receptor (Supplementary Fig. 4D). These observations taken together with data presented in Fig. 2c,d confirm that the complexes of ^Inact^β_2_V_2_R^phos^ and ^Act^β_2_V_2_R^phos^ with βarr1 represent, partially engaged (‘tail only') and fully engaged (‘tail+core') complexes, respectively. It is interesting to note here that we observe a decrease in bimane fluorescence but not a shift in emission *λ*_max_. This indicates that the decrease in bimane fluorescence most likely arises from quenching by a tyrosine or a tryptophan residue on the receptor and not directly from a different environment sensed by the bimane fluorophore^22^.

### Core interaction is dispensable for ERK2 binding

**Figure 4:**
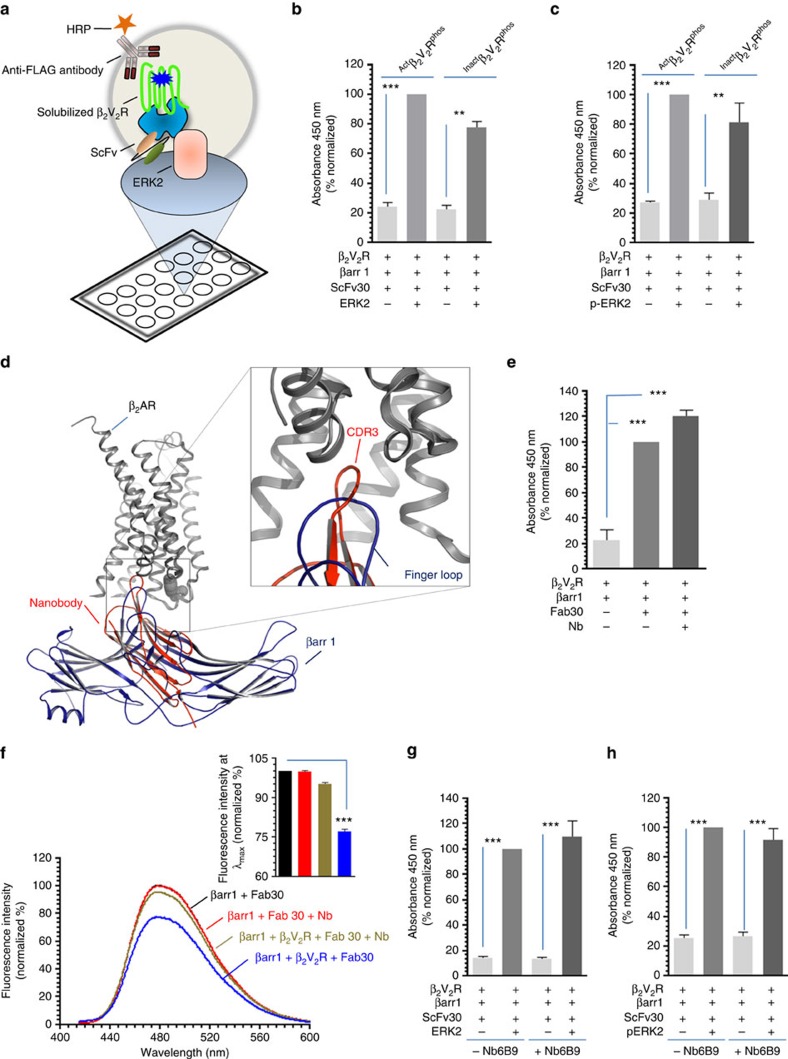
Core interaction is dispensable for recruitment of ERK2 MAP kinase. (**a**) An ELISA based approach to test the interaction of purified ERK2 with pre-formed β_2_V_2_R–βarr1-ScFv30 complex. Purified ERK2 (inactive or active) is immobilized on polystyrene surface followed by incubation with either the ‘tail only' engaged or ‘fully' engaged pre-formed complex. Interaction of ERK with the complex is visualized using HRP-coupled anti-FLAG M2 antibody as a read out of β_2_V_2_R retention on the plate. (**b**) Both ‘tail only' engaged (^Inact^β_2_V_2_R^phos^+β-arr1+ScFv30) and ‘fully' engaged (^Act^β_2_V_2_R^phos^+β-arr1+ScFv30) complexes interact with immobilized inactive (non-phosphorylated) ERK2. (**c**) Similar to inactive ERK2, phosphorylated ERK2 (that is, active) also interacts with both, the ‘tail only' engaged and ‘fully' engaged complexes. (**d**) A previously described conformationally selective nanobody (Nb6B9) against agonist bound β_2_AR conformation has an overlapping interface with the core interaction. Structural representation based on superimposition of crystal structure of agonist bound β_2_AR and nanobody Nb6B9 (PDB ID:4LDO) and electron microscopy based model of β_2_V_2_R–βarr1 complex. (**e**) Pre-incubation of ^Act^β_2_V_2_R^phos^ with purified Nb6B9 does not affect its physical interaction with βarr1. Purified ^Act^β_2_V_2_R^phos^ was first incubated with a threefold molar excess of Nb6B9 and subsequently used for the assembly of β_2_V_2_R–βarr1-Fab30 complex in ELISA format. (**f**) Pre-incubation of ^Act^β_2_V_2_R^phos^ with Nb6B9 abolishes bimane fluorescence quenching observed upon interaction with βarr1 suggesting that presence of Nb6B9 in ^Act^β_2_AR^phos^+βarr1+Fab30 complex converts it to ‘tail only' engaged complex. (**g**) Interaction of inactive ERK2 and (**h**) active ERK2 with Nb6B9 stabilized ‘tail only' engaged complex as assessed by ELISA, further suggests that the core interaction is dispensable for ERK recruitment. Data presented in **b**, **c**, **e**, **g** and **h** represent mean±s.e.m. of three independent experiments each carried out in duplicate and analysed using one-way ANOVA with Bonferroni post-test ( **P<0.01; ***P<0.001).

Activation of ERK MAP kinase has been extensively used as a primary readout of βarr-dependent signalling downstream of GPCRs^23^,^24^,^25^,^26^. βarrs directly interact with ERK2 as well as upstream kinases of ERK cascade (c-Raf1 and MEK1) and it is proposed that βarrs act as scaffolds to bring the components of ERK cascade together^27^,^28^,^29^,^30^. We first measured the interaction of purified βarr1 with inactive and active ERK2 in the absence or presence of a phosphopeptide corresponding to the carboxyl terminus of the vasopressin receptor (V_2_Rpp). This phosphopeptide mimics the interaction of phosphorylated receptor tail and induces activation of βarrs^31^,^32^,^33^. We observed that βarr1 interacts efficiently with ERK2/pERK2 and this interaction is not altered significantly in the presence of V2Rpp (Supplementary Fig. 5). This finding suggests that activation of βarr per se may not be required for its interaction with ERK2 and it prompted us to hypothesize that both, ‘partially' and ‘fully' engaged complexes should be able to interact with ERK efficiently. Therefore, in order to test the functional competence of the partially engaged complex, we compared the binding of purified inactive and active ERK2 with fully engaged and partially engaged β_2_V_2_R-βarr1–ScFv30 complexes by ELISA and coimmunoprecipitation (Fig. 4a–c and Supplementary Fig. 6). Here we used an ScFv variant of Fab 30, referred to as ScFv30 (Supplementary Fig. 6A), to stabilize the β_2_V_2_R–βarr1 complex in order to minimize any potential clash with ERK binding. Similar to Fab30, ScFv30 also effectively stabilizes β_2_V_2_R–βarr1 complex (Supplementary Fig. 6B). Interestingly, as presented in Fig. 4b,c (and Supplementary Fig. 6D–G), both ^inact^β_2_V_2_R^phos^–βarr1 complex (tail engaged) and ^act^β_2_V_2_R^phos^–β-arr1 complex (fully engaged) exhibited robust binding to inactive (non-phosphorylated) and active (phosphorylated) ERK2. These data directly suggest that the core interaction in β_2_V_2_R–βarr1 complex is dispensable for ERK binding. We note that the interaction of ERK2 MAP kinase with ^act^β_2_V_2_R^phos^–βarr1 complex is slightly higher than ^inact^β_2_V_2_R^phos^–βarr1 complex in the ELISA format and this observation perhaps reflects relatively higher stability of the agonist bound quaternary complex under the experimental conditions.

In order to further corroborate these findings, we utilized a previously described nanobody (referred to as Nb6B9) that selectively recognizes agonist bound β_2_AR conformation and represents a G protein mimetic^34^. CDR3 of this nanobody displays a significantly overlapping interface on the receptor with that of the finger loop of βarr1 (Fig. 4d). Therefore, we reasoned that pre-incubation of this nanobody with ^Act^β_2_V_2_R^phos^ should preclude the finger loop mediated core interaction with βarr1. We first confirmed that binding of Nb6B9 to β_2_V_2_R does not affect the assembly of β_2_V_2_R–βarr1-Fab30 complex (Fig. 4e). We then tested the effect of Nb6B9 on bimane fluorescence in β_2_V_2_R–βarr1-Fab30 complex. As presented in Fig. 4f, indeed pre-incubation of this nanobody to the receptor followed by addition of βarr1 and Fab30 abolished bimane fluorescence quenching that is observed in the absence of this nanobody. This data suggests that Nb6B9 blocks the core interaction between the β_2_V_2_R and βarr1. Interestingly, however, the presence of this nanobody does not affect the interaction of the complex with active and inactive ERK2 MAP kinase (Fig. 4g,h). This observation taken together with the data presented in Fig. 4b,c confirms that the core interaction in β_2_V_2_R–βarr1 complex is dispensable for ERK binding.

### Core interaction is dispensable for ERK activation

**Figure 5:**
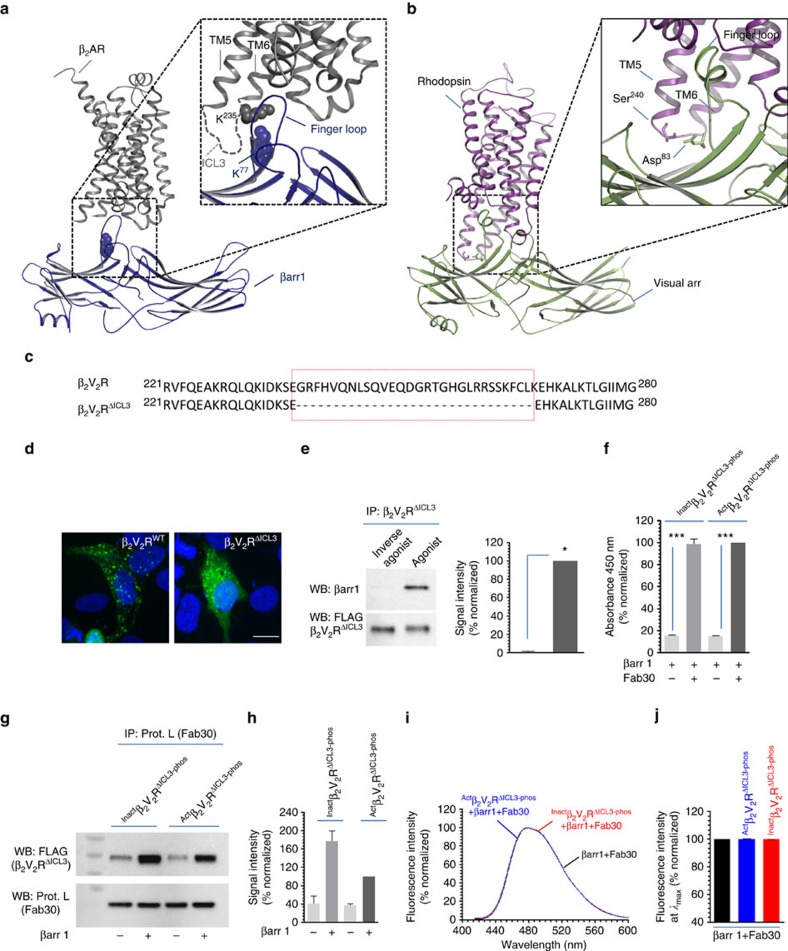
Truncation of the third intracellular loop in β_2_V_2_R ablates core interaction with βarr1. (**a**) Cross-linking experiments and electron microscopy based structural model of β_2_V_2_R–βarr1 complex has identified the third intracellular loop of the β_2_V_2_R as prominent interface for core interaction through docking of the finger loop of βarr1. Residues that are identified to cross-link with each other in β_2_V_2_R–βarr1 complex are labelled and their side chains are highlighted as space fill model. (**b**) Cross-linking studies and X-ray crystal structure of rhodopsin-visual arrestin also displays the vicinity of the third intracellular loop in rhodopsin with the finger loop of visual arrestin. (**c**) Sequence alignment of β_2_V_2_R and β_2_V_2_R^ΔICL3^ (third intracellular loop truncated receptor) to highlight the deleted amino acids (Gly^238^-Lys^267^) (red box). (**d**) Confocal microscopy of HEK-293 cells expressing either β_2_V_2_R or β_2_V_2_R^ΔICL3^ with β-arr1-YFP. Agonist stimulation leads to accumulation of endocytotic vesicles that indicates recruitment of βarr1 to activated receptor. Nuclear staining is shown using 4,6-diamidino-2-phenylindole. Compared with β_2_V_2_R, β_2_V_2_R^ΔICL3^ exhibits somewhat weaker recruitment of βarr1 as reflected by less punctate appearance. Scale bar, 10 μm. (**e**) Coimmunoprecipitation of β_2_V_2_R^ΔICL3^ with βarr1 expressed in HEK-293 cells further confirms the recruitment of βarr1 to the truncated receptor upon agonist stimulation. Cells were stimulated with agonist (Isoproterenol, 10 μM for 30 min at 37 °C) followed by cross-linking using dithiobis(succinimidyl-propionate) (1 mM for 30 min at room-temperature) and subsequently, receptor–βarr1 complex was coimmunoprecipitation using anti-FLAG antibody beads. (**f**) Assembly of β_2_V_2_R^ΔICL3^+β-arr1+Fab30 complex as measured using ELISA approach and (**g**) coimmunoprecipitation experiment. Similar to β_2_V_2_R, β_2_V_2_R^ΔICL3^ also forms a stable complex with βarr1 in the presence of Fab30. (**h**) Quantification of β_2_V_2_R^ΔICL3^–βarr1 complex formation as assessed by coimmunoprecipitation. (**i**) Bimane fluorescence spectroscopy on β_2_V_2_R^ΔICL3^ complex reveals the absence of fluorescence quenching even in the presence of agonist and thereby suggests the lack of core interaction. (**j**) Bimane fluorescence at emission *λ*_max_ as measured in **i** is presented as bar graph. Data in **f** represents mean±s.e.m. of three independent experiments each carried out in duplicate and analysed using one-way ANOVA with Bonferroni post-test (***P<0.001). Data in **g** and **h** represent two independent experiments.

We next tested whether β_2_V_2_R engaged to βarr1 only through the tail interaction is sufficient to trigger ERK activation in cells. As mentioned earlier, chemical cross-linking and structural modelling has identified the third intracellular loop in β_2_V_2_R as a major site for the core interaction with βarr1 (Fig. 5a). In particular, Lys^235^ on the third intracellular loop of β_2_V_2_R cross-links with Lys^77^ in the finger loop of βarr1 (Fig. 5a, inset). Furthermore, cross-linking studies and recent crystal structure of rhodopsin-visual arrestin complex has also identified the third intracellular loop as a part of the interface for the core interaction (Fig. 5b). Therefore, we generated a truncated β_2_V_2_R construct that harbours deletion of the third intracellular loop (Δ239−267; referred to as β_2_V_2_R^ΔICL3^) (Fig. 5c). Agonist stimulation of HEK-293 cells expressing β_2_V_2_R^ΔICL3^ leads to significant recruitment of βarr1, albeit somewhat weaker than β_2_V_2_R, as assessed by confocal microscopy (Fig. 5d) and coimmunoprecipitation experiment (Fig. 5e). This data suggest that the absence of the third intracellular loop and, therefore, the core interaction does not ablate βarr1 binding to the activated receptor in cellular context. In order to further confirm the interaction of β_2_V_2_R^ΔICL3^ with βarr1 and the status of core interaction in its complex with βarr1, we expressed and purified β_2_V_2_R^ΔICL3^ using baculovirus infected *Sf*9 cells (Supplementary Fig. 3). As presented in Fig. 5f–h, purified β_2_V_2_R^ΔICL3^ formed a stable complex with βarr1 in the presence of Fab30 as evaluated by ELISA and coimmunoprecipitation experiments. Most interestingly, β_2_V_2_R^ΔICL3^ even in the presence of agonist (that is, ^Act^β_2_V_2_R^ΔICL3-phos^) did not exhibit any bimane fluorescence quenching upon interaction with βarr1 (Fig. 5i,j), indicating the inability of β_2_V_2_R^ΔICL3^ to engage the core interaction with βarr1.

**Figure 6:**
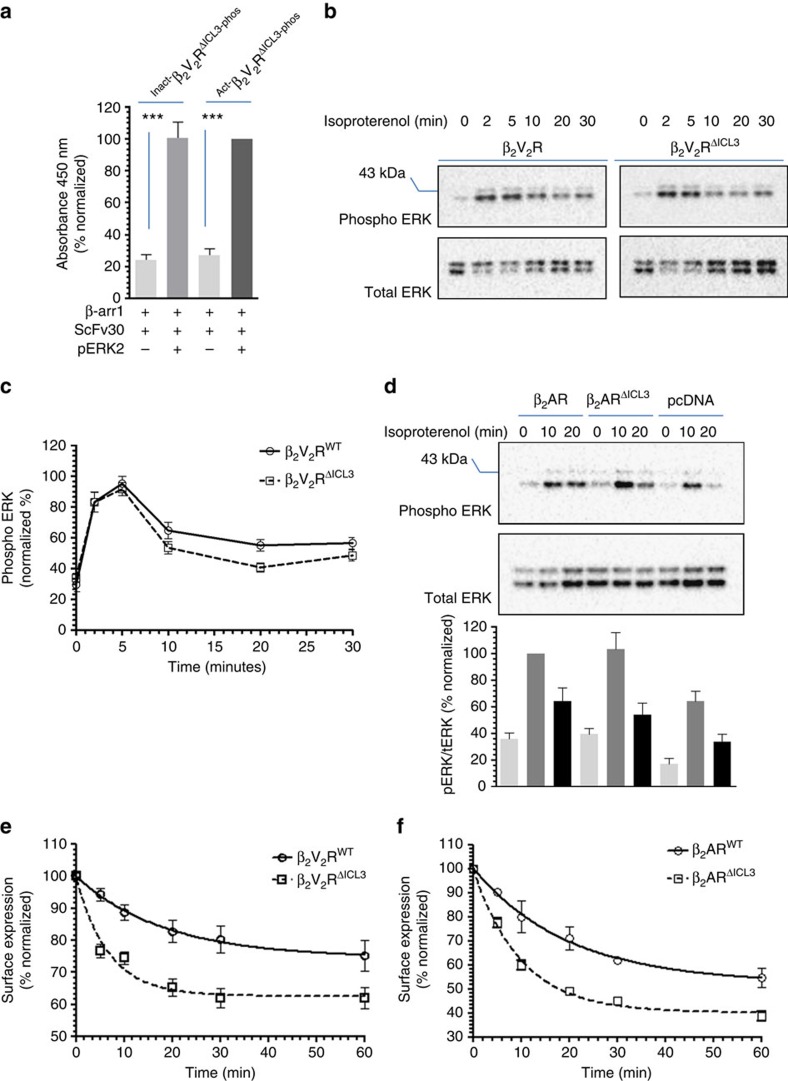
Truncation of the third intracellular loop does affect ERK activation and internalization. (**a**) Interaction of phosphorylated ERK2 MAP Kinase with β_2_V_2_R^ΔICL3^+βarr1+ScFv30 complex as assessed by ELISA approach. Similar to β_2_V_2_R, β_2_V_2_R^ΔICL3^ also forms stable complexes with phosphorylated ERK2. Data represent mean±s.e.m. of three independent experiments each carried out in duplicate and analysed using one-way ANOVA with Bonferroni post-test (***P<0.001). (**b**) Agonist induced activation of ERK1/2 MAP kinase for β_2_V_2_R and β_2_V_2_R^ΔICL3^ shows a similar temporal pattern suggesting that truncation of the third intracellular loop, and, therefore, ablation of the core interaction, does not significantly affect ERK activation. The experiment was repeated four times with identical results and a representative image is shown. (**c**) Quantification of the ERK activation data presented as mean±s.e.m. of four independent experiments. (**d**) Agonist induced activation of ERK1/2 MAP kinase downstream of β_2_AR^WT^ and β_2_AR^ΔICL3^ also reveals similar pattern suggesting the dispensability of the core interaction even for class A receptors. A representative image and quantitation of seven independent experiments are shown. (**e**) Similar to ERK activation, agonist induced internalization of β_2_V_2_R^ΔICL3^ also exhibits a comparable pattern to β_2_V_2_R^WT^ albeit with an increased kinetics. (**f**) β_2_AR^ΔICL3^ also undergoes robust internalization upon agonist stimulation similar to β_2_AR^WT^. Data in **e** and **f** represent six independent experiments each carried out in duplicate and presented as mean±s.e.m.

In order to further confirm the dispensability of the core interaction for ERK recruitment, we probed whether a complex of β_2_V_2_R^ΔICL3^ with βarr1 can bind purified pERK2. As presented in Fig. 6a and Supplementary Fig. 7A, β_2_V_2_R^ΔICL3^–βarr1-ScFv30 complex robustly recruited pERK2 and the level of interaction was comparable to that with analogous β_2_V_2_R complex. More importantly, stimulation of cells expressing β_2_V_2_R^ΔICL3^ with agonist isoproterenol leads to robust ERK activation similar to β_2_V_2_R (Fig. 6b,c). Of particular interest is the ERK activation at late time points (10, 20 and 30 min), which are well established to be mediated by βarr-dependent and G protein independent pathway. These observations taken together with the data presented in Fig. 4 suggest that the core interaction in β_2_V_2_R–βarr1 complex is dispensable for ERK binding and activation. As mentioned earlier, the chimeric β_2_V_2_R behaves like a class B receptor with respect to βarr interaction. Therefore, in order to probe whether the core interaction might be dispensable for class A receptors as well, we generated a native β_2_AR construct with truncated third intracellular loop, referred to as β_2_AR^ΔICL3^, and measured agonist induced ERK activation. Interestingly, we found that similar to β_2_V_2_R, truncation of the third intracellular loop in native β_2_AR also does not adversely affect ERK activation (Fig. 6d), suggesting that even for class A receptors, the core interaction may not be essential for stimulating ERK response.

In addition to ERK MAP kinase signalling, another key function of βarrs is to promote GPCR internalization via clathrin coated machinery^35^,^36^,^37^. It has been documented earlier that activation of βarrs with isolated V_2_Rpp leads to robust clathrin binding^32^,^33^. In fact, as presented in Fig. 5d, confocal microscopy of cells expressing β_2_V_2_R^ΔICL3^ revealed that the truncated receptor is capable of internalization as reflected by punctate appearance of βarr1-YFP upon agonist stimulation. In order to further confirm whether core interaction is dispensable for receptor internalization as well, we first measured the interaction of purified clathrin with partially and fully engaged complexes and observed comparable interaction (Supplementary Fig. 7B). In addition, we also directly compared agonist-induced internalization of β_2_V_2_R and β_2_V_2_R^ΔICL3^ by measuring surface levels of the receptor in cells. As presented in Fig. 6e, β_2_V_2_R^ΔICL3^ exhibits robust internalization upon agonist stimulation, even with slightly faster kinetics than β_2_V_2_R. Again, similar to ERK activation, we observed that β_2_AR^ΔICL3^ also undergoes robust endocytosis upon agonist stimulation (Fig. 6f). Taken together with the bimane fluorescence data, this observation suggest that both, ERK activation and receptor internalization can be efficiently supported by ‘tail only' engaged receptor–βarr complex in the absence of core interaction.

**Figure 7:**
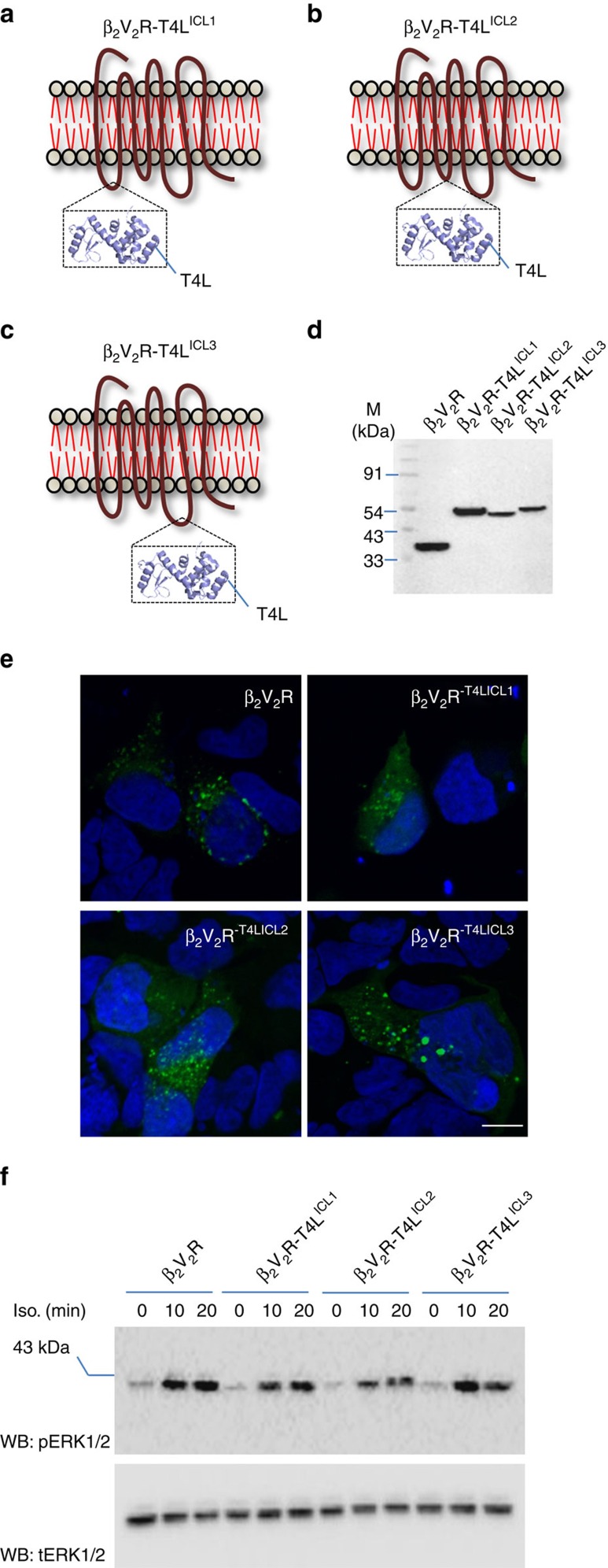
Blocking the potential contribution of intracellular loops does not affect ERK activation. Schematic illustration of β_2_V_2_R constructs with T4 lysozyme insertion in (**a**) intracellular loop 1 between Gln^65^ and Thr^66^ (**b**) intracellular loop 2 between Lys^141^ and Tyr^142^and (**c**) intracellular loop 3 between Glu^238^ and Glu^268^ with deletion of 239-267. (**d**) Expression of β_2_V_2_R-T4L constructs in transfected HEK-293 cells as visualized by western blotting using N-terminal FLAG tag. (**e**) Agonist induced βarr1 recruitment to β_2_V_2_R-T4L constructs as visualized by confocal microscopy in HEK-293 cells expressing βarr1-YFP. Scale bar, 10 μm. (**f**) Agonist (Isoproterenol, 10 μM) induced ERK1/2 activation in HEK-293 cells expressing β_2_V_2_R-T4L constructs at indicated time points. Data in **f** show a representative image of three independent experiments.

There is some evidence in the literature that the second intracellular loop, R of DRY motif in particular, of GPCRs might also contribute to receptor–βarr interaction^38^,^39^. Therefore, in order to test if ablating the potential contributions of the second intracellular loop towards the core interaction influences βarr recruitment and signalling, we inserted T4 lysozyme in the second intracellular loop of the β_2_V_2_R (between Lys^141^ and Tyr^142^; construct referred to as β_2_V_2_R-T4L^ICL2^) (Fig. 7a–d). We reasoned that the bulky T4 lysozyme would separate the receptor core from βarr through steric hindrance while not affecting βarr1 recruitment through the phosphorylated tail. We also tested in parallel β_2_V_2_R constructs with T4L in the first intracellular loop (T4L inserted between Gln^65^ and Thr^66^; β_2_V_2_R-T4L^ICL1^) and third intracellular loop (T4L inserted between Glu^238^ and Glu^268^ with deletion of 239-267; β_2_V_2_R-T4L^ICL3^) (Fig. 7a–d). As presented in Fig. 7e, all these constructs exhibited βarr1 recruitment to the receptor upon agonist stimulation as evaluated by confocal microscopy. More interestingly, these constructs also supported agonist induced ERK activation in cells similar to β_2_V_2_R and, therefore, indicate that the lack of potential contributions of first and second intracellular loops towards core interaction can also be tolerated for ERK activation.

### A β-arrestin biased ligand does not promote core interaction

**Figure 8:**
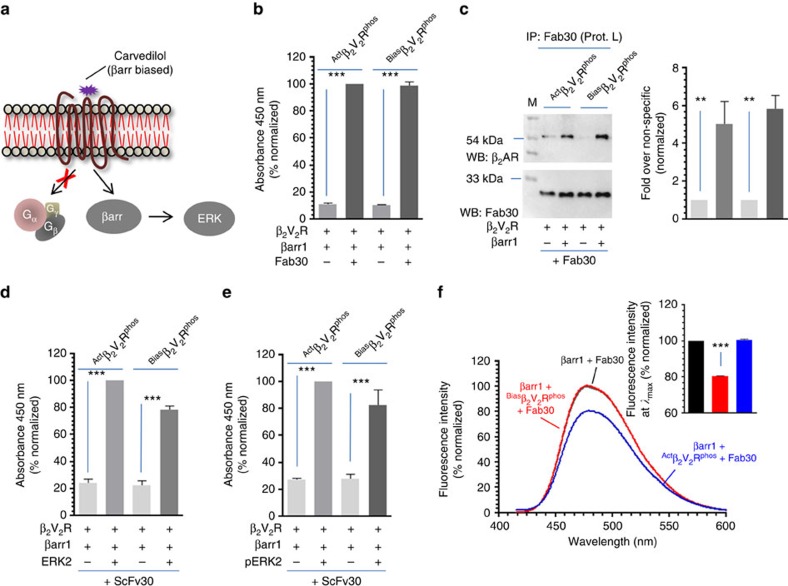
A βarr biased ligand of β_2_AR does not promote core interaction with βarr1. (**a**) Carvedilol is a high-affinity βarr biased ligand of β_2_AR and it selectively promotes βarr binding and ERK activation in the absence of any detectable G protein coupling. (**b**) Carvedilol bound and phosphorylated β_2_V_2_R (referred to as ^Bias^β_2_V_2_R^phos^generated through incubation of ^Apo^β_2_V_2_R^phos^ with tenfold molar excess of carvedilol) exhibits a robust interaction with βarr1 in the presence of Fab30 as assessed by ELISA. Purified Fab30 was immobilized and then incubated with βarr1 and either ^Bias^β_2_V_2_R^phos^ or ^Act^β_2_V_2_R^phos^. Formation of complex was detected using anti-FLAG M2 antibody. (**c**) Formation of βarr1 complex with ^Bias^β_2_V_2_R^phos^ in the presence of Fab30 as assessed by coimmunoprecipitation. The experiment was repeated three times with identical results and a representative image is shown. Quantification of the data is shown as bar graph. (**d**) Interaction of ^Bias^β_2_AR^phos^+βarr1+ScFv30 and ^Act^β_2_V_2_R^phos^+βarr1+ScFv30 complexes with inactive and (**e**) active ERK2. Purified ERK2 was immobilized followed by incubation with pre-formed complexes and detection using HRP-coupled anti-FLAG M2 antibody. (**f**) Interaction of ^Bias^β_2_V_2_R^phos^ with βarr1 does not lead to a detectable decrease in bimane fluorescence suggesting the lack of core interaction. ^Apo^β_2_V_2_R^phos^ was first incubated with tenfold molar excess of carvedilol or BI-167107 to obtain ^Bias^β_2_V_2_R^phos^ and ^Act^β_2_V_2_R^phos^, respectively. Subsequently, these receptor preparations were incubated with bimane labelled βarr1 and Fab 30 to form a complex followed by fluorescence scanning in the wavelength range indicated on the graph. The data represent an average of three independent experiments. Data presented in **b**, **d** and **e** represent mean±s.e.m. of at least three independent experiments each carried out in duplicate and analysed using one-way ANOVA with Bonferroni post-test (**P<0.01; ***P<0.001).

An interesting avenue in GPCR signalling that has emerged recently is the concept of biased agonism^40^,^41^ and for several GPCRs, biased ligands are described that selectively trigger one or the other signalling pathways downstream of the receptor^42^. For perfectly biased βarr biased ligands, there is no coupling of heterotrimeric G proteins and, therefore, no requirement of steric hindrance based desensitization of G protein signalling. We, therefore, hypothesized that a βarr biased ligand may not promote core engagement between the receptor and βarr. Carvedilol has been described as a high-affinity βarr biased ligand for β_2_AR and it promotes βarr interaction and ERK activation in the absence of any detectable G protein coupling^43^ (Fig. 8a). Carvedilol occupied β_2_V_2_R (referred to as ^Bias^β_2_V_2_R^phos^) exhibited a robust interaction with βarr1 as assessed by ELISA (Fig. 8b) and coimmunoprecipitation (Fig. 8c). Furthermore, ^Bias^β_2_V_2_R^phos^–βarr1-Fab30 complex also displayed robust interaction with inactive and active ERK (Fig. 8d,e). Most interestingly, the interaction of ^Bias^β_2_V_2_R^phos^ with bimane labelled βarr1 did not result in any detectable quenching of bimane fluorescence (Fig. 8f). These findings indicate that in response to a βarr biased ligand, receptor and βarr might engage only through the phosphorylated carboxyl terminus without any significant involvement of the core interaction.

## Discussion

Agonist activation results in a conformational change in GPCRs which in turn leads to heterotrimeric G protein coupling and downstream responses. Activated receptors are phosphorylated by GRKs which then promotes the recruitment of βarrs. It is generally believed that binding of βarrs to GPCRs sterically precludes further G protein coupling leading to receptor desensitization^44^,^45^. In fact, superimposition of β_2_AR–G protein complex crystal structure^46^ with electron microscopy based architecture of β_2_AR–βarr1 complex^11^ reveals a significantly overlapping interface on the receptor for βarr1 and the Gαs (Supplementary Fig. 8A). Moreover, crystal structure of rhodopsin with Gα C terminus peptide (GαCT)^47^ and arrestin finger loop peptide^9^ has revealed overlapping binding sites for the G protein and arrestin on the intracellular surface of the receptor. These observations indeed support steric hindrance based desensitization mechanism through competition for an overlapping interface on the cytoplasmic surface of the receptor. Interestingly, negative stain EM analysis of the β_2_V_2_R–βarr1 complex revealed a stable intermediate state in the biphasic interaction that represents a complex between β_2_V_2_R and βarr1 associated solely through the phosphorylated carboxyl terminus of the receptor^11^. Stable isolation and direct visualization of this partially engaged complex underscores the sufficiency of phosphorylated receptor tail for a physical complex formation with βarr and hints at its potential functional significance. Interestingly, crystal structure of pre-activated visual arrestin^48^ and V_2_Rpp bound βarr1^31^ have revealed major conformational changes compared with basal arrestin conformation. These changes include ∼20 Å movements of the N- and the C-domain relative to each other and disruption of the polar core. These observations suggest that even partially engaged arrestin might be primed and conformationally competent to initiate at least some of βarr functions. Our data presented here indeed suggest that partially engaged β_2_V_2_R–βarr1 complex associated only through the carboxyl terminus is sufficient to bind both, inactive and active ERK2. Furthermore, a truncated β_2_V_2_R lacking the 3rd intracellular loop and thereby defective in making core interaction with βarr not only recruits βarr1 in cells but also results in agonist stimulated ERK activation and receptor internalization. Considering these findings, it is tempting to suggest that the core interaction between the GPCR and βarrs might be essential for desensitization through steric hindrance while the tail interaction is sufficient, at least for some of the functional outcomes such as ERK binding, activation and receptor internalization (Supplementary Fig. 8B).

Based on their relative patterns of βarr recruitment, GPCRs are broadly categorized as either class A or class B receptors^18^. Class A receptors, such as β_2_AR, bind transiently to βarrs and show rapid recycling to the cell surface after internalization. Class B receptors on the other hand, such as V_2_R, exhibit a more robust interaction with βarrs and show proteosomal degradation. Class B receptors typically harbour phosphorylatable Ser/Thr clusters in their carboxyl terminus while class A receptors appear to primarily have more scattered Ser/Thr residues. It is conceivable that such clusters of Ser/Thr in class B receptors impart a stronger cumulative contribution towards higher affinity for βarrs. Two recent studies using FlAsH based βarr2 sensors suggest distinct conformational signatures of βarr2 imparted by class A vs class B GPCRs^49^,^50^. Although we have primarily used a chimeric receptor, β_2_V_2_R that displays class B profile of βarr recruitment, we also demonstrate that even for a prototypical class A GPCR, β_2_AR, core interaction is not essential for ERK activation and internalization. This observation indicates that both, class A and B receptors are capable of undergoing endocytosis and triggering ERK activation when engaged with βarrs only through the phosphorylated carboxyl terminus. Along similar lines, a recent investigation has documented that βarr2 can mediate ERK activation downstream of β_1_AR despite a very transient interaction and dissociation from the receptor^51^,^52^. Going forward, it would be interesting to test additional receptor systems to evaluate the generality of these observations in a broader context.

Constitutive activity of GPCRs refers to the basal level of activation even in the absence of activating ligand. For a number of GPCRs, constitutive activity has been detected with respect to G protein activation and it is thought to arise from the abilities of the receptors to sample active like conformations even in the absence of activating ligands. Here we observe that there is a small but significant core interaction between the Apo-receptor and βarr as assed by bimane fluorescence spectroscopy (Fig. 3), which is destabilized or stabilized by the incubation of this complex with inverse agonists or agonists, respectively. These findings raise the possibility that some basal level of βarr recruitment might exist in cells even in the absence of stimulating ligand and in fact may be responsible for desensitizing the constitutive receptor activity and some basal level of βarr signalling. Future investigations will be required to carefully probe this aspect of GPCR signalling.

It is important to mention that βarrs mediate and regulate multiple functions downstream of GPCRs. For example, βarrs can scaffold the components of clathrin mediate internalization machinery such as clathrin and AP2 and have a key role in GPCR internalization^35^,^53^. In addition to ERK MAP kinase, βarrs also scaffold components of other MAP kinase pathways (such as JNK^54^,^55^, p38) as well as c-Src^56^ and Akt^57^. Furthermore, scaffolding of E3 ubiquitin ligases has also emerged as a new functional role of βarrs for GPCRs and non-GPCR membrane proteins^58^,^59^,^60^. Although our data suggest that βarr1 engaged to the receptor only through the phosphorylated carboxyl terminus is competent to recruit and activate ERK MAP kinase and support receptor internalization, it is plausible that core interaction might still be required for some of the other functional aspects of GPCR–βarr complex, in addition to receptor desensitization. Further investigations are required to probe such a scenario where differently engaged GPCR–βarr complexes carry out different sub-sets of functions and this might help establish a mechanistic basis for broad functional repertoire and effective functional segregation along the GPCR-βarr signalling axis. It should also be noted that even with ICL3 truncated chimeric β_2_V_2_R or with other class B GPCRs, some transient core interaction can still occur, which escapes detection in bimane fluorescence assay but might still contribute towards some of the functional outcomes.

The concept of biased GPCR signalling and development of biased ligands has refined the general understanding of receptor pharmacology^61^,^62^,^63^. For many GPCRs, biased ligands are proposed to represent better therapeutic potential over currently prescribed ones by virtue of having reduced side effects^42^. However, the mechanistic and structural insights into biased GPCR signalling remains relatively less well defined. It is proposed that biased ligands induce a distinct set of conformations in the receptor than unbiased ligands and these different conformations are subsequently recognized by downstream effectors such as βarrs^11^,^64^. As a result, effectors also adopt distinct conformations which in turn govern their functional outcome^50^,^65^. A recent study using unnatural amino acid incorporation and ^19^F-NMR on βarr1 has investigated the connection between βarr1 conformation and functional outcome^66^. This study suggests that different phosphopeptides harbouring differential phosphorylation patterns that potentially correspond to a bar-code imparted by different GRKs are capable of inducing distinct conformations in βarr1. These distinct conformations in turn fine-tune the functional outcome of βarr1 such as clathrin binding and c-Src activation^66^. Furthermore, two recent reports using βarr2 conformational sensors also suggest that not only different receptors impose different conformational signature on βarr2, but also ligands of different efficacies (such as unbiased and biased) induce detectably different conformations in βarr2^49^,^50^. However, it currently remains unknown whether a GPCR–βarr complex in response to a biased ligand is conformationally and structurally different than that in response to unbiased ligand. As βarr biased ligands selectively trigger βarr recruitment in the absence of any G protein activation, there is no requirement of desensitization of G protein signalling. Therefore, it is logical to speculate that βarr may not be required to fully engage with the receptor core. Our findings that carvedilol, a βarr biased β_2_AR ligand, does not engage core interaction between the receptor and βarr1 in fact supports such a possibility. Although carvedilol has a weak efficacy for βarr-dependent β_2_AR signalling, ^19^F NMR based analysis of carvedilol bound β_2_AR^67^ as well as chemical labelling approach^68^ has directly demonstrated that it promotes distinct conformational changes in the receptor compared with unbiased agonists or inverse agonists. However, further experimentation with other GPCRs that have more efficacious biased ligand is desirable to probe the generalization of this observation.

In conclusion, our findings reveal a previously unknown aspect of GPCR–βarr interaction and provide a potential basis for broad functional repertoire of this signalling axis. In contrast with generally anticipated notion, we demonstrate that partially engaged GPCR–βarr complex is functionally competent with respect to supporting receptor internalization, and recruitment and activation of ERK MAP Kinase. Our data also suggest that βarr biased ligands may not engage the receptor core with βarr and, therefore, identify a key mechanistic insight in to biased agonism. It would be very interesting to investigate in future whether other conserved βarr functions might also be carried out through partial engagement with the activated GPCRs.

## Methods

### General reagents and protein expression

General chemicals and cell culture consumables were purchased from Sigma-Aldrich or local vendors unless specified otherwise. Codon optimized βarr1 gene was synthesized (Genscript), sub-cloned in to pGEX4T3 vector (purchased from GE), expressed in *E. coli* (BL21) and purified using Glutathione Sepharose affinity resin^33^. Codon optimized Fab30 open reading frame was synthesized (Genscript) based on published crystal structure (PDB ID: 4JQI) (ref. 31), expressed and purified in M55244 strain of *E. coli* (purchased from American Type Culture Collection)^69^. As an alternative strategy, the coding regions for the light and heavy chains of Fab30 were cloned in pETDuet-1 vector (Novagen), expressed in BL21 (DE3) cells (NEB) with 0.5 mM isopropyl-β-D-thiogalactoside induction at 18 °C for 12–16 h (Supplementary Fig. 1). Subsequently, Fab30 was purified from total lysate on Protein L resin (purchased from GE)^69^. The coding region of nanobody Nb6B9 was synthesized based on previously published crystal structure (PDB ID: 4LDO) (ref. 34) and it was expressed in *E. coli* (Rosetta) (NEB) and purified using Ni-NTA affinity chromatography^34^. Coding region of βarr1-Cys^68^ was synthesized (Genscript) and cloned in pGEX4T3 vector followed by expression in *E. coli* (BL21) and purification on Glutathione Sepharose affinity resin (Clonetech).

Coding region of human ERK2 and constitutively active MEK1 (R4F) were synthesized (Genscript), cloned in pGEX4T3 vector and expressed in *E.coli* ShuffleT7 express cells (NEB). Protein expression was induced at OD_600_ 0.6–0.8 with 0.2 mM isopropyl-β-D-thiogalactoside at 16 °C for 12–16 h. Cell pellets were resuspended in lysis buffer (25 mM Tris-HCl, pH 7.5, 150 mM NaCl, 1 mM phenylmethyl sulphonyl fluoride, 0.25 mM dithiothreitol and lysozyme for 1 h at 4 °C. Cell suspension was sonicated, centrifuged and then loaded on to a pre-equilibrated Glutathione-Sepharose resin (GE). After overnight binding at 4 °C, beads were washed extensively and then proteins were eluted using thrombin protease (Sigma or Merck). For ERK2 phosphorylation, a reaction containing inactive ERK2 and constitutive active MEK1 (R4F) in phosphorylation buffer (20 mM HEPES, pH 7.0, 5 mM MgCl_2,_ 50 mM NaCl, 1 mM dithiothreitol, 100–200 nM ATP) was prepared and incubated for 1 h at 30 °C. The reaction was quenched by addition of stop buffer (50 mM Tris pH 7.5, 18 mM EDTA), followed by a buffer exchange step on a PD10 column. Phosphorylation of ERK2 was validated by western blotting with phospho-ERK antibody (CST, catalog number. 9101; 1:5,000 dilution).

Open reading frames of FLAG-β_2_V_2_R chimeric receptor and GRK2^CAAX^ were synthesized (Genscript) and baculovirus stocks were generated using standard protocols (Expression Systems). FLAG-β_2_V_2_R and GRK2^CAAX^ were co-expressed in *Sf*9 cells (purchased from Expression Systems) and cultured in ESF921 media (Expression Systems) and 60–66 h post-infection; cells were stimulated with indicated ligand, harvested and lysed by glass douncing. Subsequently, cells were solubilized using 0.5% (w/v) maltose neopentyl glycol (MNG, purchased from Anatrace) and purified on anti-FLAG M1 affinity resin (Sigma). Purified protein samples were either used fresh in the experiments or flash-frozen in small aliquots after addition of 10–20% glycerol and stored at −80 °C until further use.

### ELISA based assembly of β_2_V_2_R–βarr1-Fab30 complex

For ELISA based *in-vitro* assembly of β_2_V_2_R–βarr1–Fab/ScFv30 complexes, purified Fab/ScFv30 (in 20 mM Hepes, pH 7.4, 100 mM NaCl) was first immobilized on 96 well MaxiSorp polystyrene plates (Nunc) at room temperature for 1 h. Afterwards, potential non-specific binding sites in the wells were blocked by incubation with 1% BSA at room temperature for 1 h. Subsequently, mixture of ligand stimulated cell lysate (or purified receptor) was added to the wells and incubated at room temperature for 1 h. Wells were washed extensively using 20 mM Hepes, pH 7.4, 100 mM NaCl, 0.01% MNG and then incubated with 1:2,000 dilution of HRP-coupled anti-FLAG M2 antibody (Sigma, catalog number A8592). After 1 h incubation, wells were extensively washed and assembly of the complex was visualized by adding 3,3′,5,5′-tetramethylbenzidine (TMB) ELISA (Genscript or Thermo). Colorimetric reaction was stopped by adding 1M H_2_SO_4_ and absorbance was measured at 450 nm using a Victor X4 plate reader (Perkin-Elmer). All the ELISA data are normalized with respect to the signal for ^Act^β_2_V_2_R^phos^ complex which is treated as 100%.

For dephosphorylation experiment, cell lysate was incubated with *λ*-phosphatase (NEB) at 25 °C for 2 h and subsequently used for *in-vitro* assembly of the complex. Fab CTL represents a random Fab taken from the library as a negative control. For dose response ELISA experiment, different amounts of β_2_V_2_R–βarr1 mixture were added to the Fab30 coated anchor surface followed by blocking of non-specific binding surface and complex detection.

### Bimane fluorescence spectroscopy

Purified βarr1^L68C^ was buffer exchanged in 20 mM Hepes, 150 mM NaCl, pH 7.5 buffer and concentrated to ∼2.0 mg ml^−1^. It was incubated with 10-fold molar excess of monobromobimane (mBBr, Sigma-Aldrich) on ice for 1 h. Subsequently, the sample was centrifuged at 100,000*g* for 30 min to remove aggregates and then unreacted mBBr was separated on a PD10 desalting column (GE Healthcare). Labelled protein was either used in bimane fluorescence experiment right away or flash frozen with 20% glycerol for later usage. Labelling efficiency of βarr1^L68C^ under these conditions was measured to be about 85%. For fluorescence experiments, mBBr labelled βarr1^L68C^ was used at an approximate final concentration of 2 μM and it was mixed with threefold molar excess (6 μM) of purified β_2_V_2_R and Fab30 for 60 min at room temperature (25 °C). For the experiments presented in Fig. 2 and Fig. 8, purified ^Apo^β_2_V_2_R^phos^ was pre-incubated with 5–10 fold molar excess (30–60 μM) of respective ligands (30 min at 25 °C) before mixing it with βarr1 and Fab30. For the experiments presented in Fig. 3, the complex of ^Apo^β_2_V_2_R^phos^–βar1-Fab30 (6 μM:2 μM:2 μM) was allowed to form at 25 °C followed by addition of 5–10 fold molar excess of ligand (30–60 μM) and an additional 30 min incubation at 25 °C. Fluorescence scanning analysis was performed using Fluorimeter (Perkin Elmer, USA model LS-55) in photon counting mode by setting the excitation and emission band pass filter of 5 nm. For emission scan, excitation was set at 397 nm and emission was measured from 415 nm to 600 nm with scan speed of 50 nm min^−1^. Bimane fluorescence intensities in each experiment are normalized with respect to βarr1+Fab30 condition, which is treated as 100%. Fluorescence intensity was also corrected for background fluorescence from buffer and protein in all experiments and each experiment was repeated at least three times.

### ERK assay and confocal microscopy

HEK-293 cells (purchased from American Type Culture Collection) were cultured in Dulbecco's modified Eagle's complete media (Sigma) supplemented with 10% fetal bovine serum (Thermo Scientific) and 1% penicillin–streptomycin at 37 °C under 5% CO_2_. For protein expression, cells were transfected with indicated plasmids using PEI (Polyethylenimine) as the transfection reagent at a DNA to PEI ratio of 1:3 (7 μg of DNA mixed with 21 μl of PEI). Cells were serum starved for 4–12 h and then stimulated with appropriate ligands as indicated in the figure legends.

For cross-linking of β_2_V_2_R^ΔICL3^ and βarr1, Carazolol and BI-167107 stimulated HEK-293 cells were resuspended in buffer containing 20 mM HEPES pH 7.4, 100 mM NaCl, 1 × PhosStop (Roche) and 1 × complete protease inhibitor (Sigma). Cells were lysed by dounce homogenization. For cross-linking, 1 mM dithiobis(succinimidyl-propionate) (Sigma) in dimethylsulphoxide was added from 100 mM stock and lysate was tumbled at room temperature for 30 min. The reaction was quenched by adding 1M Tris buffer pH 8.0 and 1% (v/v) MNG was added for solubilization and tumbled for 3 h at 4 °C. Following solubilization, lysate was centrifuged at 21,130*g* for 30 min. The clear supernatant was collected in separate tube and freshly equilibrated M1 FLAG beads were added for immunoprecipitation. Coimmunoprecipitated βarr and β_2_V_2_R were detected by western blotting rabbit mAb anti-βarr antibody (CST, 1:1,000, catalog number D24H9) and HRP-coupled mouse anti-FLAG M2 mAb (Sigma, 1:1,000). Blots were developed on Chemidoc (Bio-Rad) and subsequently quantified by ImageLab software (Bio-Rad).

For ERK assay, transfected cells were seeded in to six-well plates (Corning), serum starved for 12 h and then stimulated with 10 μM Isoproterenol (Sigma-Aldrich) for indicated time points. Subsequently, the cells were lysed in 200 μl of 2 × SDS loading buffer, sonicated and loaded on to 12% SDS–polyacrylamide gel electrophoresis. Western blotting was performed to observe the phosphorylation of ERK1/2. The bands were transferred on PVDF membrane (BioRad). The membrane was blocked with 5% BSA (SRL) for 1 h and then probed with anti-pERK primary antibody (CST, catalog number. 9101; 1:5,000 dilution) overnight at 4 °C followed by 1 h incubation with anti-rabbit IgG secondary antibody (Genscript, catalog number. A00098) at room temperature. The membrane was then washed with 1 × TBST thrice and developed using Chemi Doc (BioRad). The anti-pERK antibody was stripped-off using 1X stripping buffer and then reprobed with anti-tERK antibody (CST, catalog number. 9102 and 4695; 1:5,000 dilution).

For confocal microscopy, transfected HEK-293 cells were seeded onto 0.001% poly-L-lysine coated glass coverslips and serum starved for 4 h. Cells were then stimulated with 10 μM Isoproterenol for indicated time points, fixed using 4% paraformaldehyde and permeabilized with 0.05% Triton-X-100. For nuclear staining, 0.5 μg ml^−1^ of 4,6-diamidino-2-phenylindole solution (Sigma) was added to fixed cells. After final washing with PBS, coverslips were mounted on to glass slides using VectaShield H-1,000 mounting medium (VectaShield), allowed to air dry for 15 min and then imaged using LSM780NLO confocal microscope (Carl Zeiss).

### Coimmunoprecipitation experiments

In order to assess the formation of β_2_V_2_R–βarr1 complex in solution by coimmunoprecipitation, purified β_2_V_2_R (2.5 μg) was mixed with purified βarr1 (2.5 or 5 μg) and Fab30 (2.5 μg) and incubated at room-temperature for 1 h. Subsequently, 20 μl of protein L beads (Capto L, GE Healthcare) were added and the mixture was allowed to tumble at room-temperature for additional 1 h. Afterwards, beads were washed three times with washing buffer (20 mM Hepes, pH 7.4, 150 mM NaCl, 0.01% MNG) and eluted with SDS loading buffer. Eluted samples were separated by 12% SDS–polyacrylamide gel electrophoresis and probed using HRP-coupled anti-FLAG M2 antibody (Sigma, 1:2,000) and HRP- coupled protein L (GenScript, 1:2,000; catalog number M00098) by western blotting.

In order to measure binding of ERK2 with pre-formed complex, purified GST-ERK2 (or GST-pERK2) (6 μg) was immobilized on freshly equilibrated GS beads (1 h at room-temperature) and washed once with washing buffer to remove unbound GST-ERK2. Subsequently, beads were incubated (1 h at room-temperature) with pre-formed β_2_V_2_R–βarr1–ScFv30 complex (4 μg:4 μg:5 μg) followed with three washes. Afterwards, bound samples were eluted in SDS loading buffer and probed by western blotting using HRP-coupled anti-FLAG M2 antibody. Purified GST was used as a control for non-specific binding of the complex to GS beads. Quantification of coIP data is normalized with respect to ^Act^β_2_V_2_R^phos^, which is treated as 100%.

### Receptor internalization assay

HEK-293 cells expressing β_2_V_2_R and β_2_V_2_R^ΔICL3^ seeded in to 24-well plates at a density of 300,000 cells per well and serum starved for 2 h. Cells were stimulated with 10 μM isoproterenol at specified time points followed by three washes with ice cold tris-buffered saline (TBS) and subsequently fixed with 4% (w/v) paraformaldehyde for 20 min on ice. Cells were again washed with TBS and blocked with TBS+1%(w/v) BSA for 1 h at room temperature. Cells were then incubated with HRP-coupled anti-FLAG M2 antibody (Sigma) at a dilution of 1:1,000 in TBS+1%BSA for 1 h at room temperature. Afterwards, cells were washed with TBS+1%(w/v) BSA three times and incubated with 200 μl 3,3′,5,5′-tetramethylbenzidine (TMB) per well for visualizing surface receptor expression. Reaction was stopped by transferring 100 μl of developed solution to a 96-well plate already containing 100 μl of 1M H_2_SO_4_. Plates were read at 450 nm in a microplate reader (Victor X4). For measuring total protein (for normalization), cells were washed with TBS and 200 μl of 0.2% (w/v) Janus green stain was added per well and incubated for 10 min. Subsequently, cells were destained with water until excess dye was removed and colour was developed by adding 800 μl of 0.5 M HCl per well. One-hundred microlitres of solution was transferred in 96-well plate and read at 595 nm in a multi-plate reader. The values were normalized by dividing A_450_ reading with A_595_ reading.

### Data analysis

All the data were plotted using GraphPad Prism software and analysed as indicated in the figure legends. For statistical analysis, we used one-way ANOVA with Bonferroni post-test. Uncropped images of key experiments are presented in the Supplementary Fig. 9.

### Data availability

The crystal structures of Fab30 and nanobody Nb6B9 bound to β-arrestin were obtained from PDB using accession codes 4JQI and 4LDO, respectively. The data that support the findings of this study are available from the corresponding author upon reasonable request.

## Additional information

**How to cite this article:** Kumari, P. *et al*. Functional competence of a partially engaged GPCR–β-arrestin complex. *Nat. Commun.* **7,** 13416 doi: 10.1038/ncomms13416 (2016).

**Publisher's note:** Springer Nature remains neutral with regard to jurisdictional claims in published maps and institutional affiliations.

## Supplementary Material

Supplementary Information Supplementary Figures 1-9.
