# Supplementary material for: Functional competence of a partially engaged GPCR–β-arrestin complex
Source: Nat Commun. 2016 Nov 9;7:13416. doi: 10.1038/ncomms13416 (PMC5105198; doi:10.1038/ncomms13416)
Supplement: Supplementary Information — Supplementary Figures 1-9. [file ncomms13416-s1.pdf]

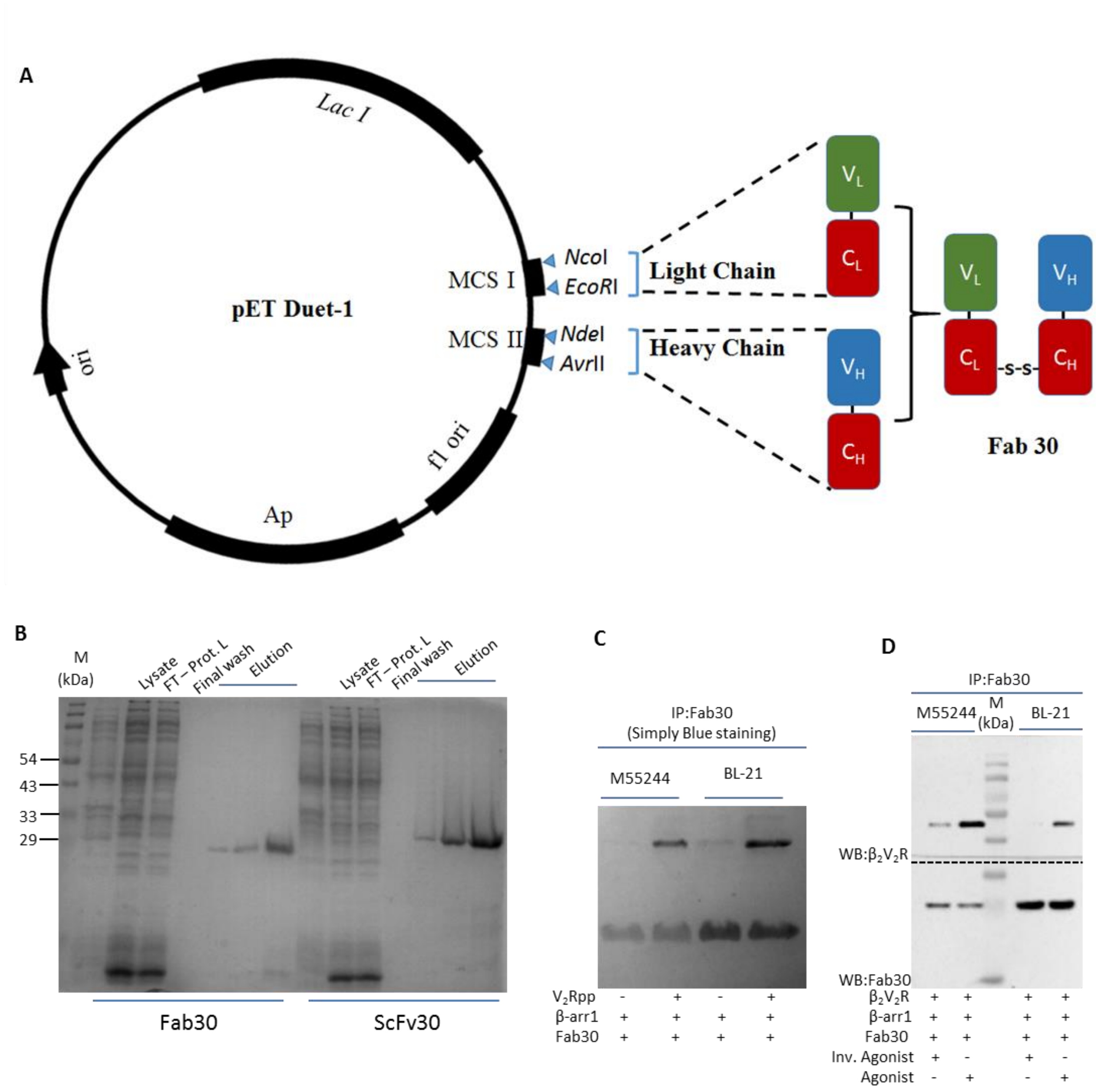

Supplementary Figure 1. An improved protocol for expression and purification of Fab30 and ScFv30. A. Coding regions of heavy and light chains of Fab30 based on previously determined crystal structure were synthesized and cloned in to bi-cistronic pETDuet-1 vector. **B.** Expression of Fab30 and ScFv30 in BL21 (DE3) cells with IPTG induction and purification on Protein L resin. **C.** Functional validation of Fab30 by its selective interaction with V_2Rpp activated β-arr1. **D.** Functional validation of Fab30 by its ability to stabilize β-arr1 complex with agonist bound and phosphorylated β_2V_2R (vs. inverse agonist bound and non-phosphorylated β_2V_2R). ScFv30 purified from BL21 cells exhibited similar functional capabilities as Fab30.

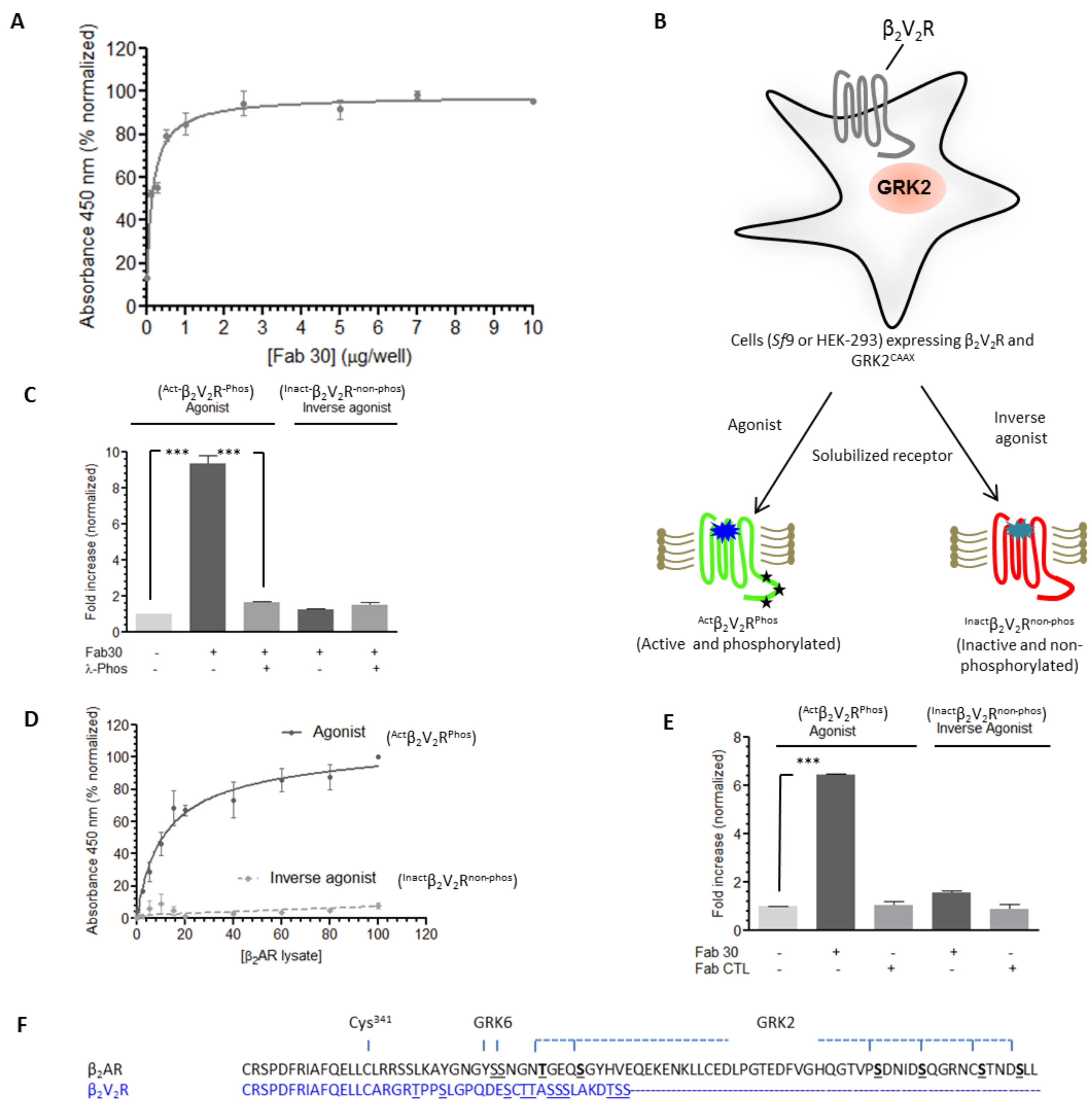

Supplementary Figure 2. Assembly of β_2V_2R-βarr1-Fab 30 complex. A. Increasing concentrations of purified Fab 30 were immobilized on to polystyrene surface and maximal immobilization was assessed by reactivity of HRP-coupled Protein L. **B.** β_2V_2R and GRK2 were co-expressed in *Sf*9 or HEK-293 cells followed by stimulation using indicated ligands. **C.** β_2V_2R-β-arr1-Fab30 complex assembly requires receptor phosphorylation as agonist bound but dephosphorylated receptor (treated with λ-phosphatase , λ-Phos.) fails to yield a detectable complex formation. **D.** β_2V_2R-β-arr1-Fab30 complex assembly is saturable and highly dependent on agonist stimulation (for receptor phosphorylatuon) of the receptor. **E.** *In-vitro* assembly of β_2V_2R- β-arr1 complex depends on Fab 30. A control Fab (Fab CTL) that does not interact with β-arrestin 1 fails to facilitate the assembly of β_2V_2R-β-arr1 complex. **F.** Sequence

comparison of the carboxyl-terminus of β_2AR and β_2V_2R (blue). Potential phosphorylation sites in β_2V_2R are underlined and known phosphorylation sites in β_2AR are in bold and underlined. Data in panels C and E represent mean±SEM of three independent experiments analyzed by ONE-WAY ANOVA with Bonferroni post-test (***$p<0.001$).

A

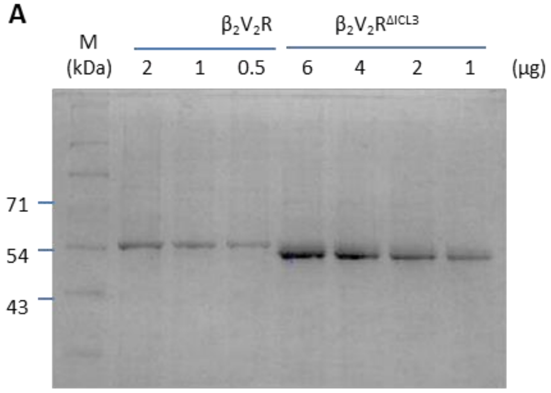

B

Construct	Average Yield
β_2V_2R	0.3-0.5 mg/L
$\beta_2V_2R^{\Delta ICL3}$	0.6-0.8 mg/L

Supplementary Figure 3. Purification of β_2V_2R and $\beta_2V_2R^{\Delta ICL3}$ using baculovirus infected *Sf*9 cells. A. Cultured *Sf*9 cells were infected with baculovirus encoding N-terminal FLAG tagged β_2V_2R (or $\beta_2V_2R^{\Delta ICL3}$) and untagged GRK2^{CAAX}. 60-66h post-infection, cells were stimulated with isoproterenol (1μM final at 37°C for 1h), solubilized using 0.5 % LMNG (2h at room-temperature) and purified by anti-FLAG affinity chromatography. Purified receptor was concentrated using a 30kDa cut-off viva-spin concentrator and the purity was analyzed by 10% SDS-PAGE stained with SimplyBlue (Invitrogen). **B.** Purification table sowing typical purification yields of β_2V_2R and $\beta_2V_2R^{\Delta ICL3}$ as measured by total protein estimation (Bradford assay) and gel-based quantification using BSA as standard.

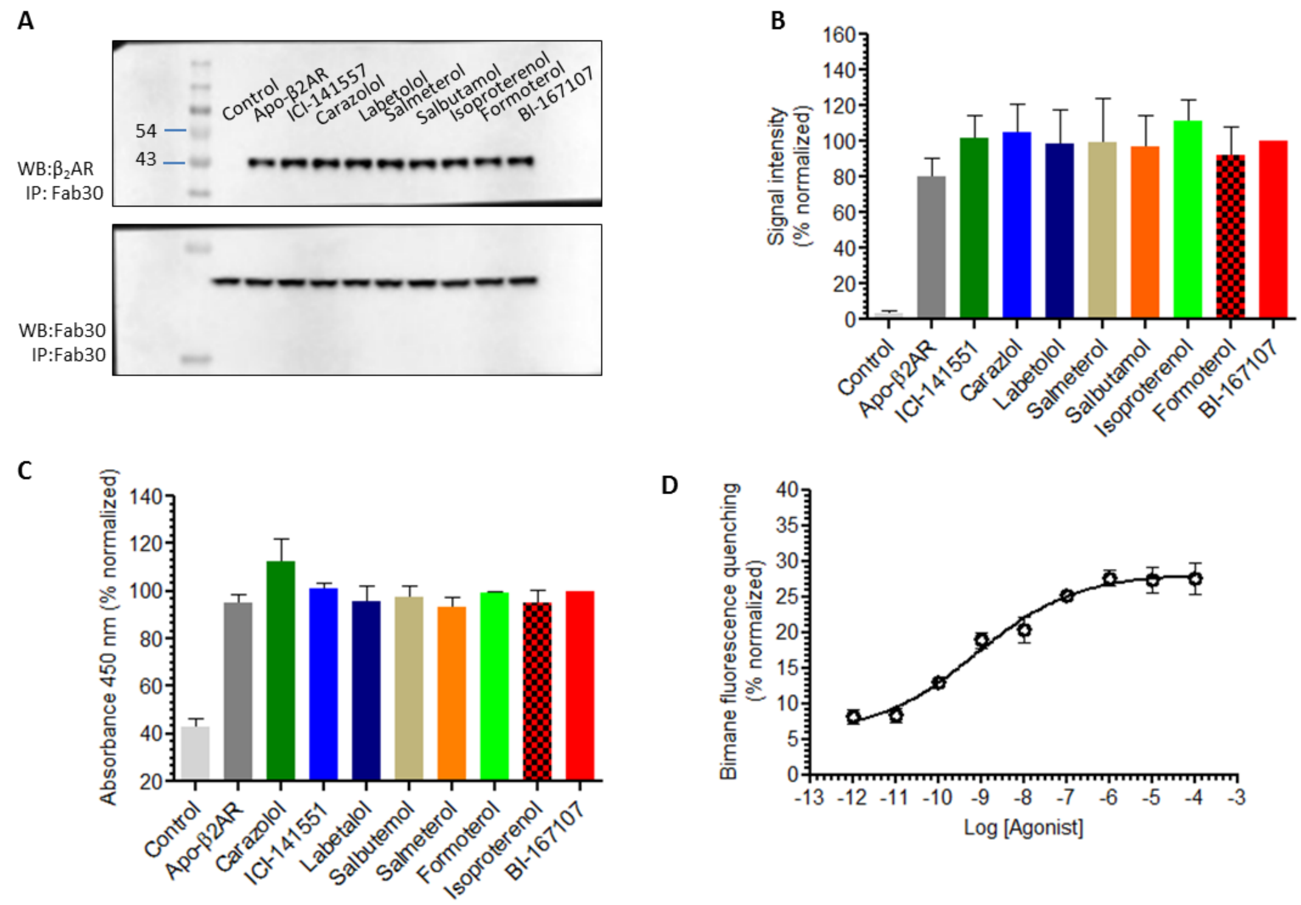

Supplementary Figure 4. *A*ssembly of β_2V_2R-βarr1–Fab 30 complex with ligands of different efficacies. A. Incubation of β_2V_2R ligands with pre-formed $^{Apo}\beta_2V_2R^{phos}$-β-arr1-Fab30 complex followed by co-immunoprecipitation reveals comparable levels of physical interaction. **B.** Quantification of data presented in panel A. **C.** Incubation of β_2V_2R ligands with pre-formed $^{Apo}\beta_2V_2R^{phos}$-β-arr1-Fab30 complex in ELISA format further confirms the comparable levels of physical interaction. **D.** Addition of varying dosage of full agonist BI-167107 leads to a pattern of bimane fluorescence quenching that directly corresponds to the agonist occupancy of the receptor. Data in panel represents mean ± SEM of three independent experiments.

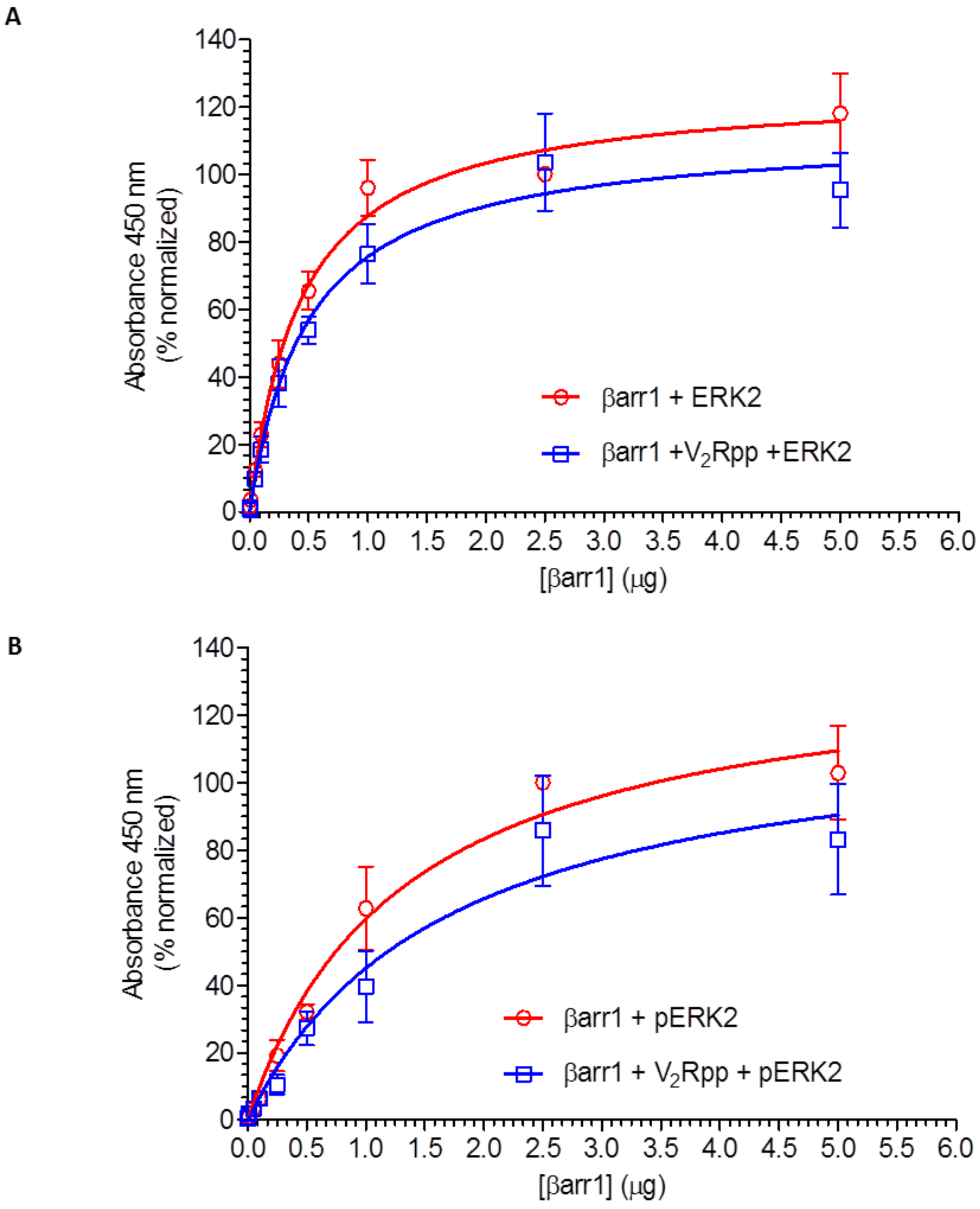

Supplementary Figure 5. Interaction of βarr1 with ERK2 in presence and absence of V_2Rpp. 2.5μg of purified ERK2 (**A**) or phosphorylated ERK2 (**B**) was immobilized in each well of a 96 well plate, followed by blocking of non-specific binding sites with 200μl of BSA (0.5%) per well. Subsequently, the wells were incubated with purified and biotinylated βarr1 (2.5μg) in presence or absence of V_2Rpp (10 fold molar excess). Post-incubation, wells were washed and incubated with HRP-coupled streptavidin followed by visualization of the signal using TMB ELISA. Data represent mean ± SEM of three independent experiments each carried out in duplicates.

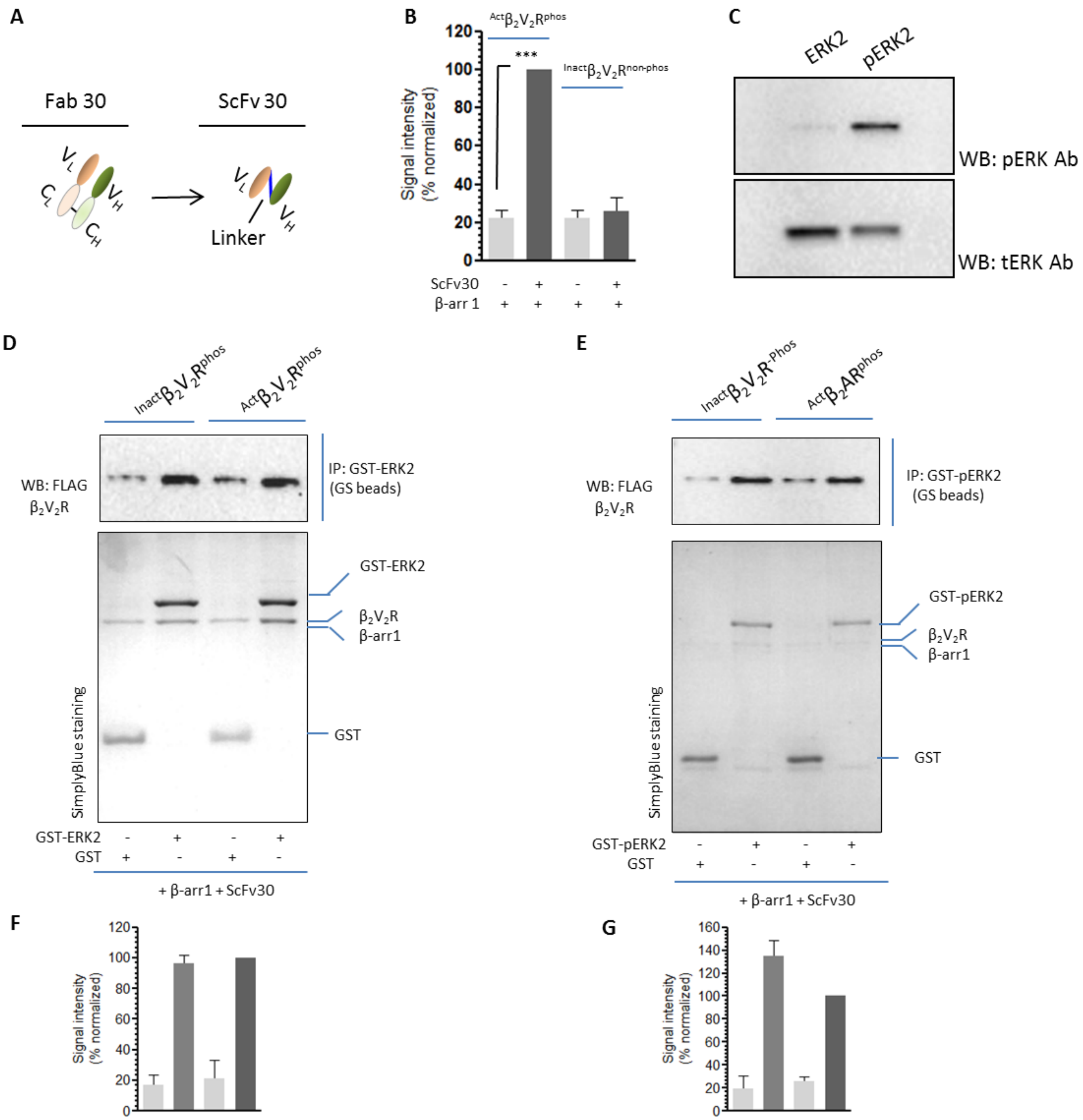

Supplementary Figure 6. Binding of β_2V_2R-βarr1–ScFv30 complexes with ERK2 MAP kinase. A. Conversion of Fab30 in to ScFv30 (single chain variable fragment) by joining the coding regions of variable light chain and variable heavy chain through a flexible linker. **B.** Purified ScFv30 also supports *in-vitro* assembly of β_2V_2R-βarr1 complex as assessed by ELISA. **C.** ERK2 MAP kinase was purified from *E. coli* and phosphorylated *in-vitro* using MEK1 as described in the Materials and Methods section. Phosphorylation status of active ERK2 was probed with anti-phospho-ERK2 antibody and anti-ERK2 antibody. **D.** Interaction of inactive ERK2 and **E.** active ERK2 with β_2V_2R-β-arr1–ScFv30 as assessed by

coimmunoprecipitation assay (representative image from three independent experiments). Quantification of complex interaction with **F.** ERK2 and **G.** pERK2. Data in panel B represent mean±SEM of three independent experiments (ONE-WAY ANOVA with Bonferroni post-test; ***p<0.001).

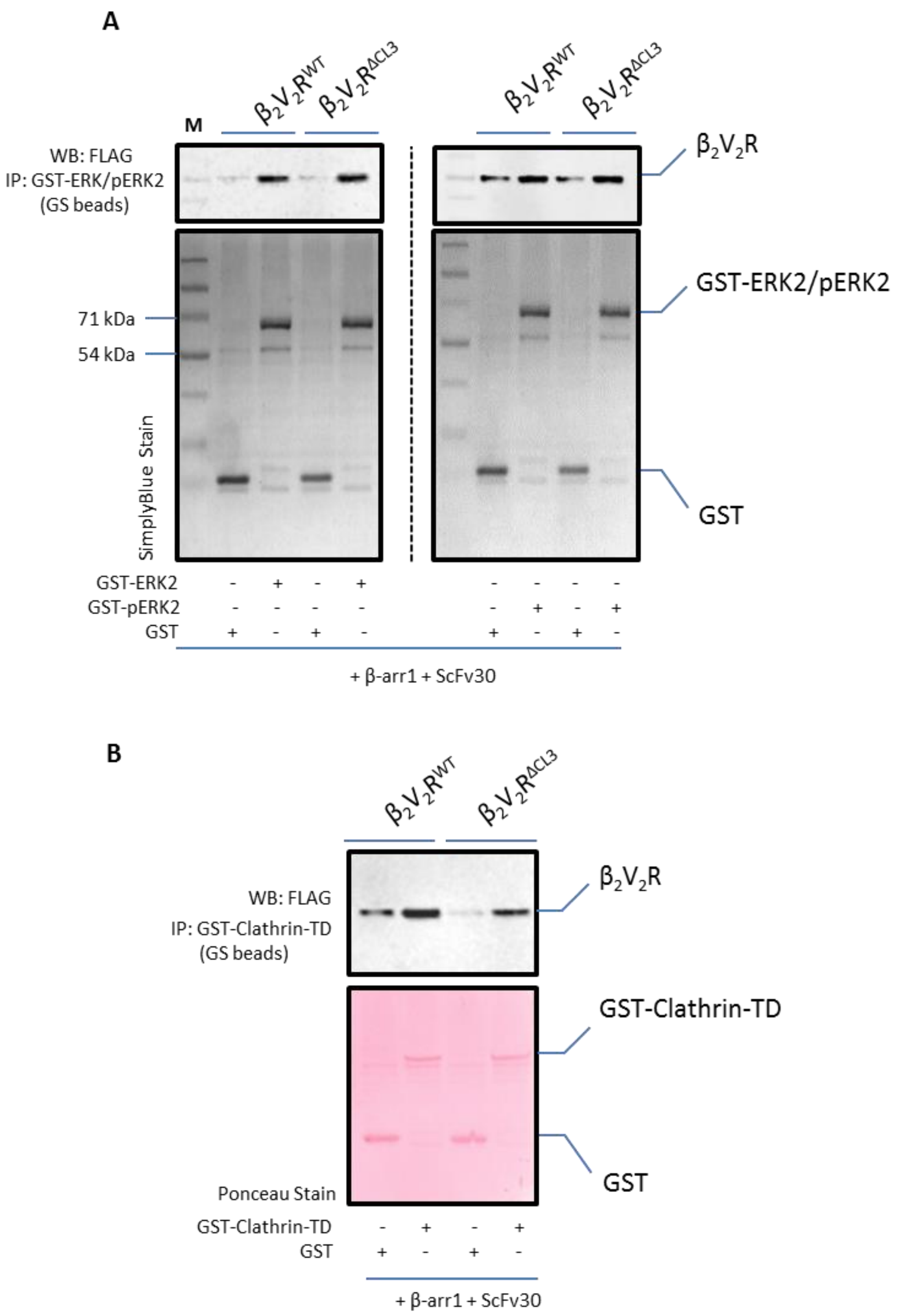

Supplementary Figure 7. Interaction of β$_2$V$_2$R-βarr1-ScFv30 complexes with ERK and Clathrin. A. Interaction of ERK2 and pERK2 with ScFv30 stabilized pre-assembled β$_2$V$_2$R^{WT}-βarr1 and β$_2$V$_2$R$^{\Delta ICL3}$-βarr1 complexes. These complexes represent "fully engaged (core +tail) and partially engaged ("tail only") complexes, respectively, and they both are capable of recruiting inactive (non-phosphorylated) and active (phosphorylated) ERK2. **B.** Interaction of purified Clathrin-TD (Terminal Domain) with ScFv30 stabilized pre-assembled β$_2$V$_2$R^{WT}-βarr1 and β$_2$V$_2$R$^{\Delta ICL3}$-βarr1 complexes. A aepresentative image of three independent experiments is shown.

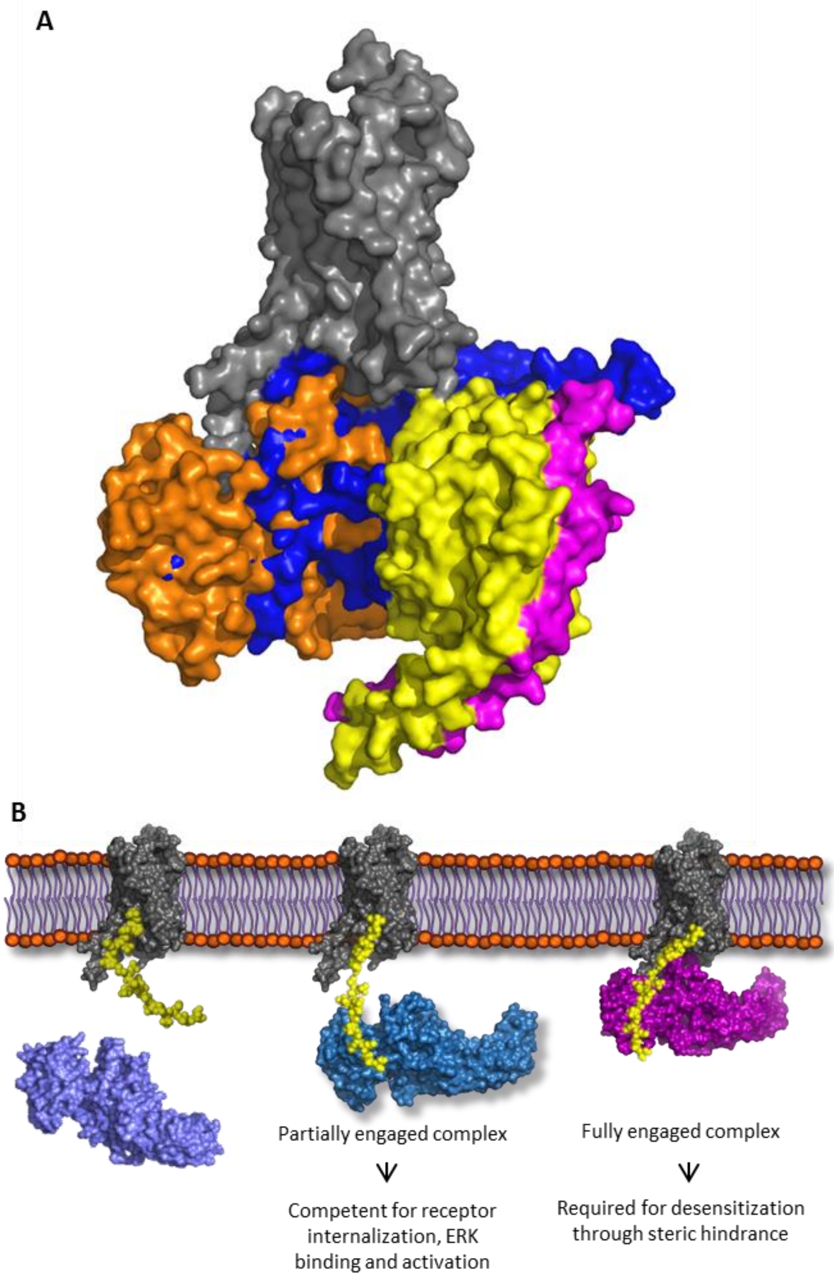

Supplementary Figure 8. A model depicting the functional competence of partially engaged complex. **A.** Superimposition of β_2AR-G protein crystal structure (PDB ID: 3SN6) with EM based structural model of β_2V_2R-βarr1 complex reveals an overlapping interface of the Gαs and the βarr1 on the receptor. β_2AR is in grey, Gαs is in blue, Gβ is in yellow, Gγ is in pink and β-arrestin 1 is in orange. **B.** A schematic illustration to propose that the tail engaged complex is sufficient for receptor internalization, ERK binding and activation while fully engaged complex is required for receptor desensitization.

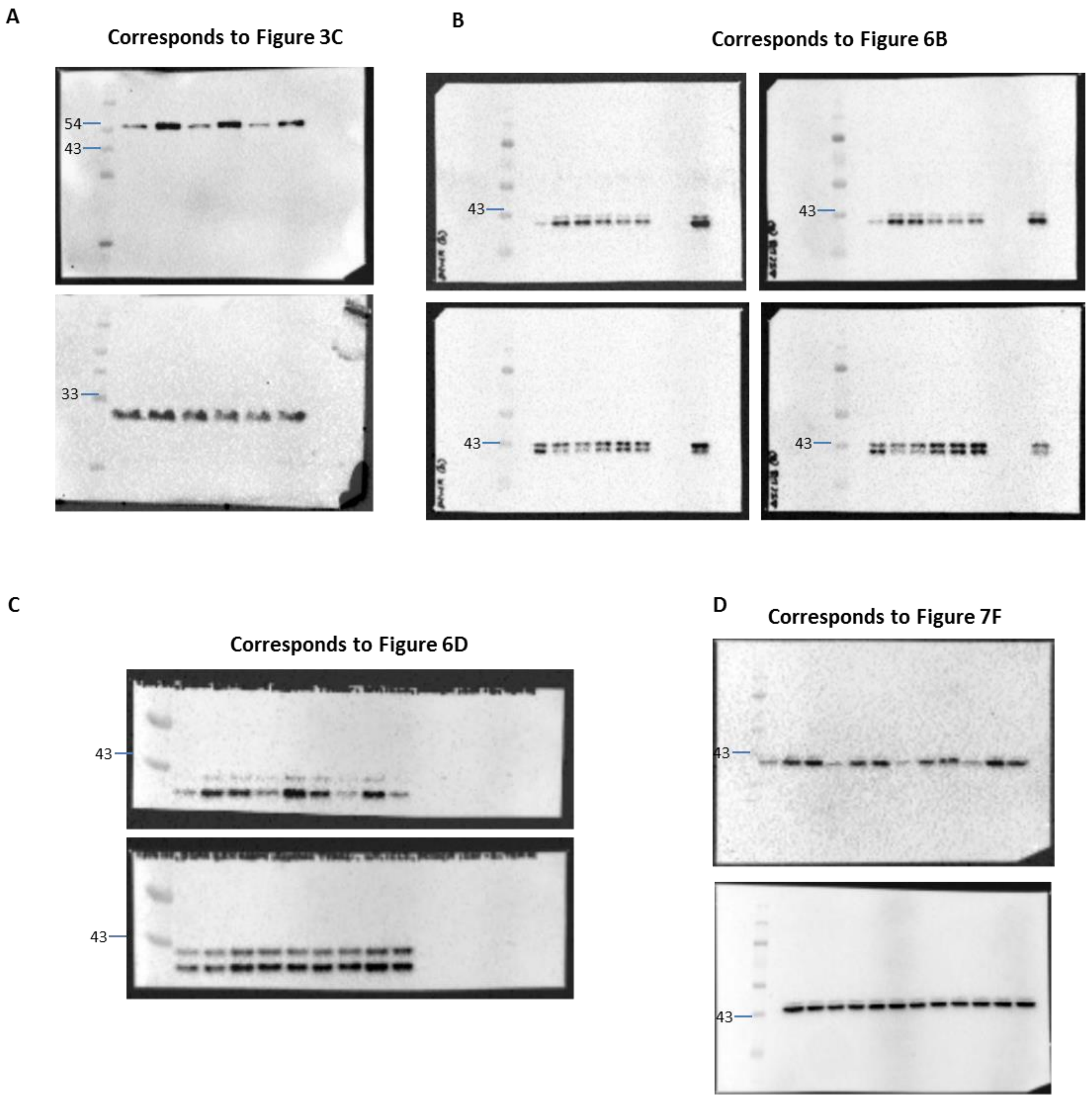

Supplementary Figure 9. Full images of the key Western blot data presented in the manuscript. Corresponding main text figures are indicated above the images. Merged images with molecular weight markers are presented.
